# Dynamics of alkannin/shikonin biosynthesis in response to jasmonate and salicylic acid in *Lithospermum officinale*

**DOI:** 10.1038/s41598-022-21322-0

**Published:** 2022-10-12

**Authors:** Muhammad Ahmad, Alicia Varela Alonso, Antigoni E. Koletti, Nebojša Rodić, Michael Reichelt, Philipp Rödel, Andreana N. Assimopoulou, Ovidiu Paun, Stéphane Declerck, Carolin Schneider, Eva M. Molin

**Affiliations:** 1 Center for Health & Bioresources, AIT Austrian Institute of Technology GmbH, Tulln, Austria; 2 Institut für Pflanzenkultur GmbH & Co. KG., Schnega, Germany; 3 Earth and Life Institute, Université Catholique de Louvain, Louvain-la-Neuve, Belgium; 4 Department of Chemical Engineering, Laboratory of Organic Chemistry and Center of Interdisciplinary Research and Innovation, Natural Products Research Centre of Excellence (NatPro-AUTh), Aristotle University of Thessaloniki, Thessaloníki, Greece; 5 Department of Biochemistry, Max Planck Institute for Chemical Ecology, Jena, Germany; 6 Department of Botany and Biodiversity Research, University of Vienna, Vienna, Austria

**Keywords:** Plant hormones, Secondary metabolism

## Abstract

Alkannin/shikonin and their derivatives are specialised metabolites of high pharmaceutical and ecological importance exclusively produced in the periderm of members of the plant family Boraginaceae. Previous studies have shown that their biosynthesis is induced in response to methyl jasmonate but not salicylic acid, two phytohormones that play important roles in plant defence. However, mechanistic understanding of induction and non-induction remains largely unknown. In the present study, we generated the first comprehensive transcriptomic dataset and metabolite profiles of *Lithospermum officinale* plants treated with methyl jasmonate and salicylic acid to shed light on the underlying mechanisms. Our results highlight the diverse biological processes activated by both phytohormones and reveal the important regulatory role of the mevalonate pathway in alkannin/shikonin biosynthesis in *L. officinale*. Furthermore, by modelling a coexpression network, we uncovered structural and novel regulatory candidate genes connected to alkannin/shikonin biosynthesis. Besides providing new mechanistic insights into alkannin/shikonin biosynthesis, the generated methyl jasmonate and salicylic acid elicited expression profiles together with the coexpression networks serve as important functional genomic resources for the scientific community aiming at deepening the understanding of alkannin/shikonin biosynthesis.

## Introduction

Alkannin, its (R)-enantiomer shikonin and their derivatives (altogether herein denoted as A/S), are a group of bioactive specialised metabolites (SMs), so far known to be mainly produced by members of the Boraginaceae family^1^. These red/purple pigmented compounds are biosynthesized in the cytosol and accumulated in the apoplastic space of the root periderm^1^. Historically, periderm extracts of *Alkanna* and *Lithospermum* species have been used as dyes and crude drugs in European and Asian countries, respectively^2^. Currently, A/S are used in cosmetic, food and pharmaceutical industries based on their broad spectrum of biological activities^2,3^. Very recently, shikonin has been described as an inhibitor of the main protease of SARS-CoV-2^4,5^. In addition, the inhibitory effects of A/S on fungi and the strong induction of A/S biosynthesis in response to phytopathogens, drought stress and elevated temperature suggest that A/S are important defence-related compounds^6–8^. Since A/S are released into the rhizosphere^9^, they have also been suggested to mediate plant-plant^7^ and plant–microbe interactions^10^.

**Figure 1:**
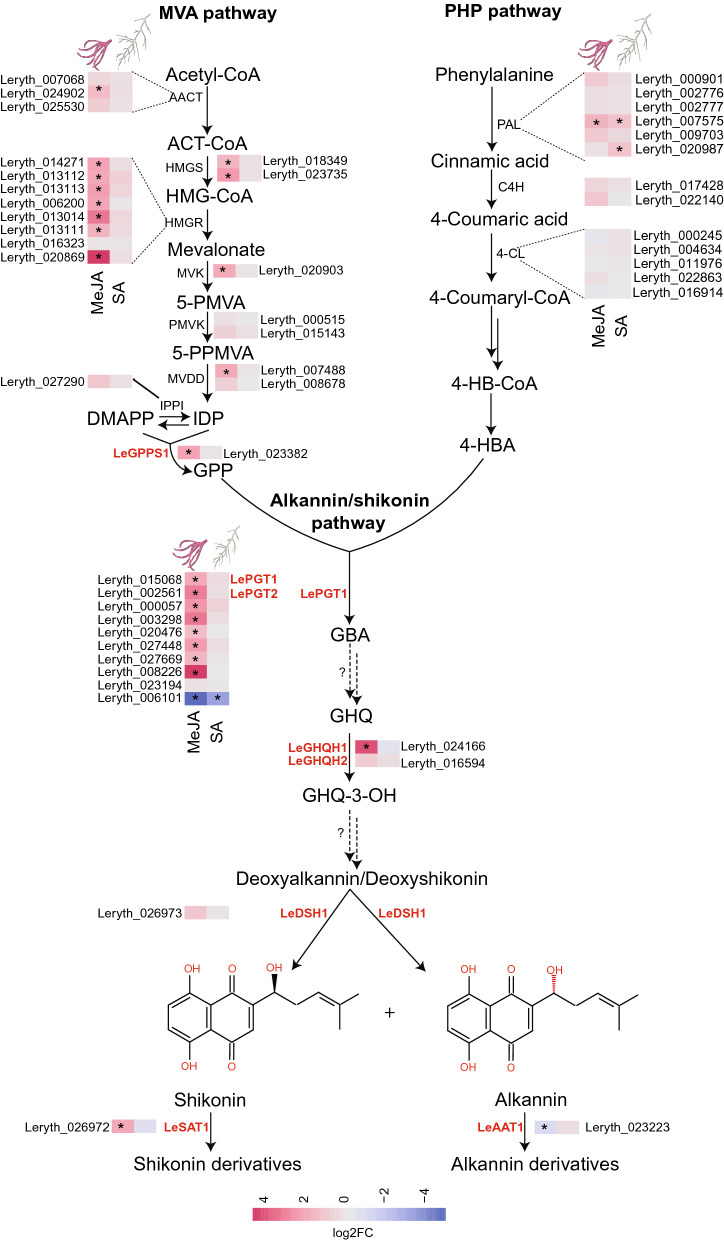
The current understanding of the alkannin/shikonin (A/S) biosynthetic pathway, together with the effect of methyl jasmonate (MeJA) and salicylic acid (SA) on gene expression. For differential expression, raw reads were mapped to *Lithospermum erythrorhizon* genome, a sister species to *L. officinale* with very recent divergence histories (divergence time approx. 0.5 million years). Uncharacterized steps in the pathway are shown with question marks and dashed arrows. Single and double dashed arrows depict one and multiple steps that are unknown, respectively. Coloured letters indicate characterised steps. The mean log2 fold-change in expression level of MeJA (red) and SA (white) treated roots as compared to control in mevalonate (MVA), phenylpropanoid (PHP), and A/S pathway are shown. *Denotes differentially expressed genes at log2fold >|1| and FDR < 0.05. Abbreviations: HMGR, 3-hydroxy-3-methylglutaryl-CoA reductase; HMG-CoA, 3-hydroxy-3-methylglutaryl-CoA; HMGS, 3-hydroxy-3-methylglutaryl-CoA synthase; MVK, mevalonate 5-phosphokinase; 5-PMVA, 5-phosphomevalonate; PMVK, 5-phosphomevalonate phosphokinase; 5-PPMVA, 5-diphosphate mevalonate; MVDD, mevalonate diphosphate decarboxylase; IDP, isopentenyl diphosphate; IPPI, isopentenyl pyrophosphate isomerase; DMAP, Dimethylallyl diphosphate; GPPS, Geranyl diphosphate synthase; GPP, Geranyl diphosphate; PAL, phenylalanine ammonia lyase; C4H, cinnamic acid 4-hydroxylase; 4-CL, 4-Coumarate ligase; 4-HB-CoA, 4-hydroxybenzoyl-CoA; 4-HBA, 4-hydroxybenzoic acid. PGT, 4-hydroxybenzoate-3-geranyltransferase; GHQ, geranylhydroxyquinone, GHQH, geranylhydroquinone 3′′-hydroxylase; GHQ-3-OH, 3-hydroxy-geranyl hydroxy quinone; DSH, deoxyshikonin hydroxylase; SAT, shikonin acyl transferase; AAT, alkannin acyl transferase.

Due to the great industrial and potential ecological significance of A/S, several studies have been devoted to deciphering their biosynthetic pathway^11–15^. However, the pathway is still not completely elucidated. A/S biosynthesis is built on both, the phenylpropanoid and mevalonate pathway (reviewed in^1^), which are then joined through the fusion of *4*-hydroxybenzoic acid (4-HBA [phenylpropanoid]) and geranyl diphosphate (mevalonate)^1^ for the specific and final part of the biosynthesis route (Fig. 1). Genes of the mevalonate pathway have already been well described^16^. Conversely, in the phenylpropanoid pathway, the genes involved in the last steps leading to 4-HBA remain unknown^16^. After biosynthesis of 4-HBA and geranyl diphosphate, these intermediates are coupled together by the action of *p*-hydroxybenzoate: geranyltransferase (LePGT1;^1^) to produce the first product of the A/S pathway (3-geranyl-4-hydroxybenzoic acid [GBA]). GBA is either decarboxylated and/or hydroxylated to produce geranyl hydroquinone (GHQ;^16^). However, the catalysing enzymes and corresponding genes involved in these steps remain to be discovered. What is known is that GHQ further undergoes hydroxylation by cytochrome P450 to produce GHQ-3-OH that might subsequently be catalysed in several steps to yield deoxy-A/S by unknown enzymes^17^. In the final step, deoxy-A/S is hydroxylated by CYP82AR-like enzymes to yield A/S^18^. However, the genes corresponding to this step and the previous ones have not yet been functionally characterized. Meanwhile, except for LeMYB1 and LeEIL-1^19,20^, no other transcription factors regulating A/S biosynthesis have been characterized.

Under biotic and abiotic stresses, plants synthesise SMs, a process that is often tightly regulated by phytohormones^21^. Among them, jasmonic acid (JA) or its methyl derivative (MeJA), indole-3-acetic acid and ethylene have been shown to induce A/S biosynthesis^1,22^. In contrast, salicylic acid (SA) either does not affect A/S biosynthesis^23^ or—likely similar to abscisic acid—negatively regulates it^24,25^. Phytohormone-induced differential biosynthesis of A/S has already been demonstrated in cell cultures^22,23^. However, knowledge transfer to whole plant systems is not straightforward, since already several differences have been described in the A/S biosynthesis between cell cultures and hairy root systems (reviewed in^1^).

Therefore, the main objective of the present study was to investigate the roles of JA and SA on A/S biosynthesis using a recently developed whole plant *Lithospermum officinale *in vitro cultivation system^26^, and to identify candidate structural and regulatory genes of the A/S biosynthesis pathway. Towards this aim, we first quantified A/S levels in response to MeJA and SA confirming earlier observations from cell culture studies, and then we generated transcriptomic data for roots of *L. officinale* plants treated with MeJA and SA against controls to provide new insights into the molecular mechanisms of A/S accumulation. We further developed a coexpression network to pinpoint potential synthetic and novel regulatory genes of the A/S pathway in *L. officinale*.

## Results

### A/S and phytohormonal profiles of *L. officinale* roots following MeJA and SA treatment

**Figure 2:**
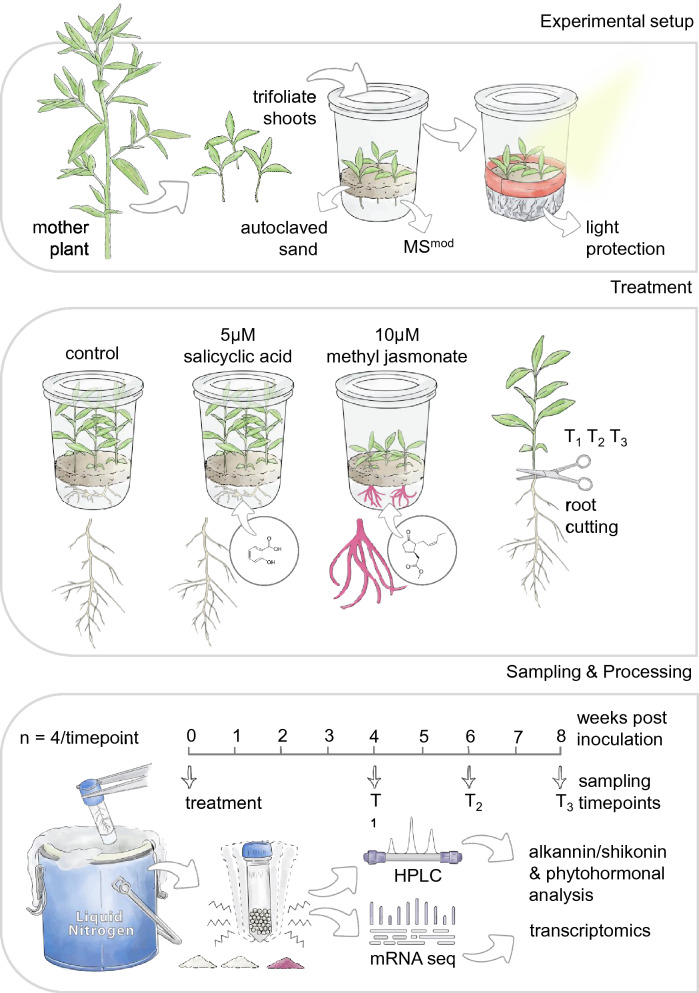
Study system, experimental setup and sampling scheme to investigate the effect of MeJA and SA on A/S biosynthesis in *Lithospermum officinale*. Shoot cuttings of *L. officinale* were transferred to MSR modified medium supplemented with MeJA or SA. The roots and shoots of three to five replicates were harvested at 4 weeks post inoculation (wpi), 6 wpi and 8 wpi, and were used for metabolite analysis and mRNA sequencing. Designed by Tatjana Hirschmugl (https://scillustration.at/en/).

**Figure 3:**
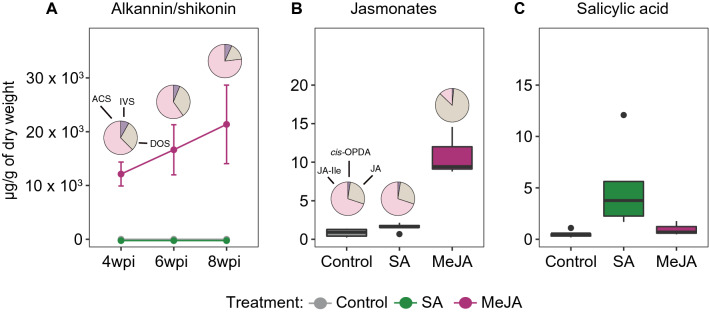
Effect of MeJA and SA on the biosynthesis of alkannin/shikonin (A/S) and phytohormones. (**A**) Levels of total A/S (line graph) and proportion (pie plot) of different A/S derivatives quantified in roots of *L. officinale* after 4 weeks post inoculation (4 wpi), 6 wpi and 8 wpi of respective treatments. (**B**) Total jasmonates (boxplot) and proportion (pie plot) of different derivatives of jasmonates. (**C**) SA in the roots of *L. officinale* after 8 wpi. n = 3–5 replicates for each treatment and each time point. Abbreviations: ACS, Acetyl-A/S; DOS, Deoxy-A/S; IVS, Isovaleryl-A/S, JA-Ile, Jasmonate-isoleucine; *cis*-OPDA, cis-Oxophytodionic acid; JA, jasmonic acid.

To assess the effect of MeJA and SA on the A/S profile, we quantified A/S in the roots of plants treated with the respective hormone at 4, 6, and 8 weeks post inoculation (wpi; for experimental setup details see Fig. 2). A/S was at the limit of detection of the chromatograph and could neither be detected in the roots of the plants from the SA treatment nor in the corresponding control at all three time points (Fig. 3A). In contrast, in the MeJA treatment, total A/S contents (sum of all derivatives) reached up to 21 × 10^3^ µg/g of dried root weight (Fig. 3A). Although a much higher accumulation of total A/S contents at the final time point of the MeJA treatment was observed compared to earlier time points (Fig. 3A), the differences over time within MeJA treatment were not significant (*p* ≥ 0.29). Finally, among all derivatives detected in the roots of MeJA treated plants, acetyl-A/S showed higher levels of accumulation followed by deoxy-A/S and isovaleryl-A/S (Fig. 3A), while A/S and *β, β*–dimethylacryl-A/S were present only in trace amounts (data not shown).

To understand which phytohormones accumulated differentially under A/S-producing (MeJA treatment) and non-producing conditions (SA and control treatments), the hormonal profiles of *L. officinale* roots were analysed at 8 wpi. The spectrum of quantified hormones included abscisic acid, indole-3-acetic acid, SA, JA, the conjugate jasmonoyl isoleucine and the JA precursor 12-oxophytodienoic acid. As expected, the levels of total jasmonates (sum of all jasmonates) increased significantly (*p* < 0.001) under the MeJA treatment as compared to the SA and control treatments (Fig. 3B). Among these jasmonates, JA itself was the most abundant one followed by JA-isoleucine and 12-oxophytodienoic acid in the MeJA treatment (Fig. 3B). In SA treated plants, only SA itself was observed to be accumulated (Fig. 3C). No other significant differences were noticed for the other tested phytohormones (data not shown). Taken together, these results suggest that the biosynthesis of A/S and its derivatives are mostly a response to MeJA application, accompanied by an increased accumulation of jasmonates.

### MeJA induced important shifts in the transcriptional profiles of *L. officinale* roots

To investigate A/S biosynthesis and system-wide shifts in root transcriptional profiles in response to MeJA and SA, a comparative transcriptomics analysis was conducted on root samples using four replicates at three time points (4, 6 and 8 wpi, cf. Figure 2), except for the MeJA treatment at 8 wpi, for which only three replicates were available. Overall, mRNA sequencing yielded an average of 33.6 million read pairs per sample. After preprocessing, 93% of the raw reads could be retained for downstream analysis (Supplementary Table S1). Since no genome was available for the studied species, we mapped high quality reads to the genome of *L. erythrorhizon* (version 1.0)*,* a closely related sister species of *L. officinale* with an estimated divergence time of ~ 0.5 million years^27,28^*.* The overall alignment rate was 91% (~ 28 million reads per sample), of which a higher proportion of high-quality reads mapped uniquely (85%) and to the exonic regions (81%; Supplementary Table S2).

**Figure 4:**
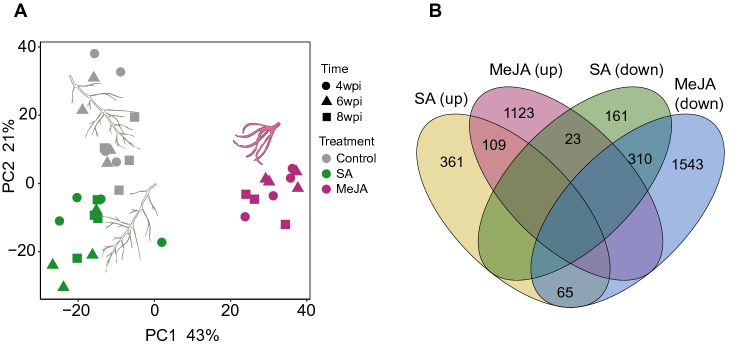
Effect of MeJA and SA on the root transcriptomes in *Lithospermum officinale*. (**A**) Principal component analysis of transcriptional variation of phytohormone treated roots. (**B**) Common and unique up- and down-regulated differentially expressed genes between MeJA and SA treatments in comparison to control conditions.

Principal component analysis showed that the transcript profiles of all samples could be classified into three distinct categories matching the respective MeJA, SA, and control treatments (Fig. 4A). A higher proportion of variance (43%) was explained by the separation of MeJA treated samples from the rest along the PC1 axis, while SA and control samples separated along the PC2 axis, accounting for half of the variation as compared to PC1. However, no obvious separation of samples was observed for the different time points (Fig. 4A) even at PC3 (data not shown), suggesting a similar temporal expression of transcripts within each treatment.

To gain an overall insight into transcriptomic shifts modulated by MeJA and SA, differentially expressed genes were identified as compared to the controls (Supplementary Tables S1, S2). As expected, a larger number of DEGs against controls was obtained in MeJA treated samples (1255 up- and 1918 down-regulated), as compared to the SA treatment (535 up and 494 down; Fig. 4B). Although a moderate proportion of downregulated genes were shared between MeJA and SA (8.4%, 310 genes), the upregulated genes were mainly unique to each treatment (1123 and 361 genes, respectively, Fig. 4B). Overall, these results suggest distinct expression patterns induced by MeJA and SA, where MeJA clearly had a greater impact on the modulation of transcriptional profiles of *L. officinale* roots as compared to SA.

### Biological processes regulated by MeJA and SA in *L. officinale*

**Figure 5:**
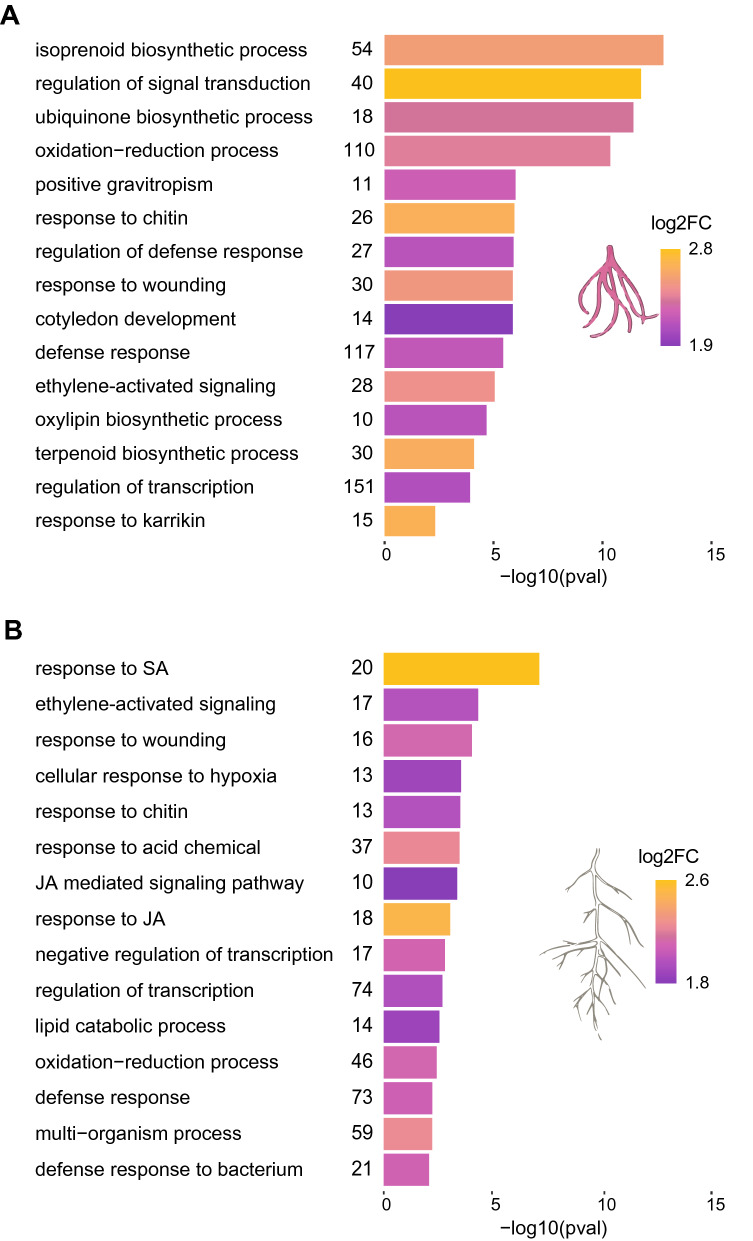
Gene ontology (GO) enrichment analysis of biological processes upregulated in response to MeJA, and SA as compared to control in *Lithospermum officinale*. Functional enrichment of (**A**) MeJA- and (**B**) SA-induced genes. Length of horizontal bars depict the degree of significance of GO terms as estimated by Fisher’s exact test. GO terms with *p*-value < 0.01 were considered overrepresented. Numbers right to the horizontal bars reflect the number of differentially expressed genes in the respective GO term. The heatmap is computed from average log2fold changes to reflect the expression of genes in the corresponding GO category. Only the top 15 (based on *p*-value) GO terms are shown here. A complete list of significant GO terms is presented in Supplementary Tables 3–6.

To understand which biological processes are modulated by MeJA and SA, gene ontology (GO) enrichment analysis of DEGs was performed. Enrichment analysis of the DEGs revealed a wide range of biological processes involved in mediating plant responses to MeJA and SA, respectively (Supplementary Tables S3–S6). Among the upregulated genes, higher-level GO categories of biological processes such as “defence response”, “regulation of transcription, DNA templated”, and “oxidation–reduction process” were associated to both treatments (Fig. 5A,B), supporting their role in the activation of the plant defence machinery. As expected, despite the occurrence of shared GO terms between MeJA and SA, these GO categories contained several distinct gene annotations (Supplementary Tables S3–S6). For example, SA treatment-related upregulated genes encompassed annotations involved in SA metabolism and regulation of SA mediated responses (Supplementary Table S4), while among MeJA treatment-related upregulated genes, GO terms had genes involved in JA biosynthesis and signalling cascades (Supplementary Table S3). In addition to the common biological processes regulated by both hormones, we observed distinct over-representations of upregulated genes associated with various metabolic pathways connected to the biosynthesis of SMs. For example, the response of MeJA treated samples specifically affected the GO terms “isoprenoid biosynthetic process”, “terpenoid biosynthetic process”, “flavonoid biosynthetic process” and “ubiquinone biosynthetic process”, which were absent in SA treated samples (Fig. 5A,B; Supplementary Tables S3, S4). On the other hand, the plants in the SA treatment changed expression of genes involved in monoterpenoid biosynthesis.

The group of downregulated genes revealed how MeJA may switch the plant’s program from growth and development to SM production and immune responses with the enrichment of GO terms such as “regulation of seedling development”, “cell wall organisation”, “cell wall biogenesis”, “xyloglucan metabolic process”, and “lipid metabolic process” (Supplementary Tables S5, S6). This is in agreement with the stunted phenotype of MeJA treated plants, where we observed up to 36% and 55% reduction in shoot length and dry weight as compared to control plants, respectively (Supplementary Fig. S1). Though not quantified, delayed root growth, decreased ramification, and less root hairs were also observed in the plants of the MeJA treatment. Notably, three of the aforementioned functional terms (“cell wall organisation”, “cell wall biogenesis”, “xyloglucan metabolic process”) were also overrepresented among SA treatment-based downregulated genes suggesting a common repressing action of MeJA and SA while regulating the respective immune responses. However, at the phenotypic level, the plants in the SA treatment showed similar growth dynamics as those in the control treatment (shoot length and dry weight, cf. Supplementary Fig. S1).

### A/S biosynthesis in response to MeJA and SA in *L. officinale*

To understand the effect of MeJA and SA on A/S biosynthesis, the expression of genes involved in precursor (mevalonate and phenylpropanoid) and A/S pathways were investigated. This analysis revealed a similar, but limited, induction of phenylpropanoid-related genes by both phytohormones (Fig. 1). For example, MeJA and SA significantly induced the expression of PAL encoding genes, a rate limiting enzyme of the phenylpropanoid pathway. Although non-significant, genes downstream of PAL (C4H and 4-CL) appeared to be similarly induced by both phytohormones. In contrast, a strong induction of genes belonging to the mevalonate pathway was only evident in the plants of the MeJA treatment (Fig. 1). For instance, transcripts of all but one copy of 3-hydroxy-3-methylglutaryl coenzyme A reductase, the rate-controlling enzyme of the mevalonate pathway, accumulated at significant levels exclusively in response to MeJA. Similarly, the expression of recently characterised cytosolic geranyl diphosphate synthase (LeGPPS1) was only significant in the plants of the MeJA treatment. LeGPPS1 catalyses the condensation of isopentenyl diphosphate and dimethylallyl diphosphate via the mevalonate route to supply the first intermediate (geranyl diphosphate) for the A/S pathway^13,29^. Geranyl diphosphate can also be derived from the plastidal methylerythritol phosphate pathway if isopentyl diphosphate and dimethylallyl phosphate exported to cytoplasm are used as substrates by LeGPPS1^13,29^. However, except for one downregulated copy of deoxy-d-xylulose-5-phosphate synthase, both MeJA and SA showed no effect on the plastidal pathway (Supplementary Fig. S2). This suggests that even under highly conducive conditions such as that of MeJA, geranyl diphosphate flux is mainly derived via the mevalonate route. In line with the increased expression of genes encoding precursors, a significantly higher expression of A/S pathway-specific genes (LePGT1, LePGT2, LeGHQH1, and LeSAT1) was noticed only in the roots of the plants of the MeJA treatment. Interestingly, apart from the characterised LePGT1 and LePGT2, other copies of PGTs (except Leryth_006101) were also significantly induced in response to MeJA highlighting the strong effect of MeJA on the A/S pathway as also observed at the metabolite level (Fig. 3).

### Weighted gene coexpression network analysis recovered known genes associated with A/S biosynthesis

**Figure 6:**
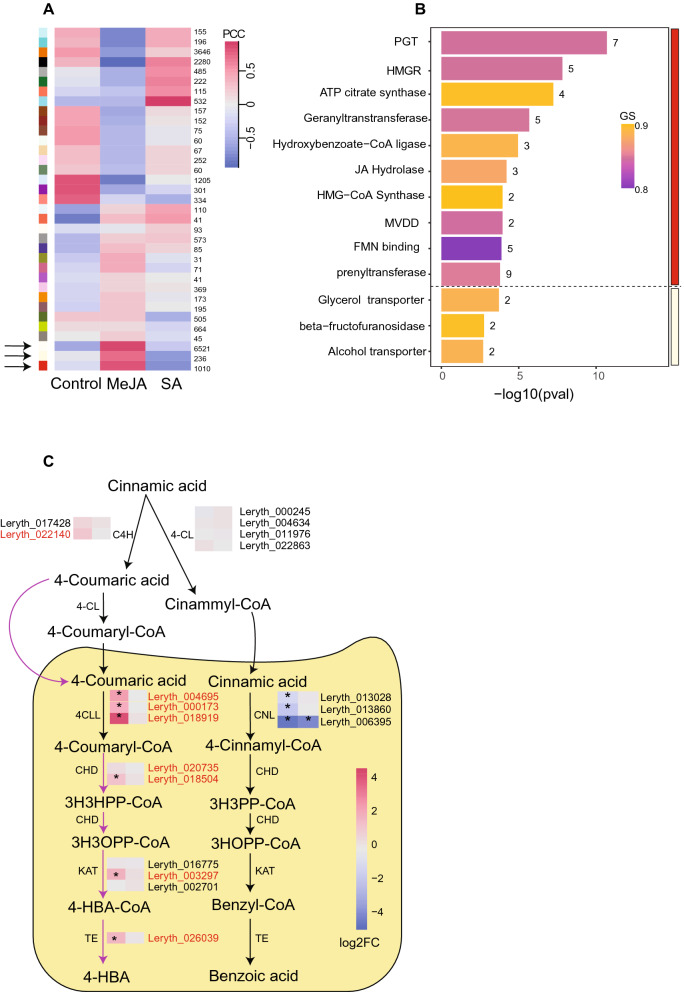
Weighted gene coexpression analysis in *Lithospermum officinale*. (**A**) Pearson correlation (PCC) of identified modules with each treatment. Each row of the heatmap depicts a single module of clustered genes with dark red and blue colours depicting strong positive and negative correlations, respectively. Numbers on the right represent the total number of genes coexpressed together in the respective module and each colour on the left represents an individual cluster of genes identified by WGCNA. (**B**) GO enrichment analysis of molecular functions of genes coexpressed with LePGT1 in the red subnetwork (n = 243) and LeSAT1 in the light-yellow module (n = 49) after filtering for gene significance (GS > 0.7) and module membership (> 0.7). The heatmap corresponds to the average GS of each GO category to the shikonin producing condition. Numbers on the right correspond to the number of genes in the respective GO term. (**C**) Expression levels of genes involved in the β-oxidative benzenoid pathway. Genes coexpressed together with LePGT1 in the red subnetwork are highlighted in red.

To identify genes that are potentially involved in A/S biosynthesis and regulation, weighted gene coexpression network analysis was performed using WGCNA v1.70-3^30^. The 21,057 genes resulting from filtering of lowly expressed genes were clustered into 35 coexpression modules (Supplementary Table S7). We hypothesised that those modules whose eigengenes (gene expression summarised as PC1) are positively correlated with the A/S-producing condition (MeJA treatment) would harbour genes related to A/S biosynthesis. Three such modules (red, ivory, and light yellow) showed highly significant correlation with the MeJA treatment (Pearson *r* = 0.75—0.88; *p* = 6.04E−05–2.03E−09; Fig. 6A; Supplementary Table S7). We only considered those genes in these modules having module memberships and gene significance ≥ 0.70. The number of retained genes in each of these subnetworks varied from 49, 243, and 1347 in the modules light yellow, red, and ivory, respectively. The red subnetwork was enriched with 54 GO categories (*p* < 0.01; Supplementary Table S8) containing genes encoding enzymes that are connected to A/S biosynthesis (Fig. 6B; Supplementary Table S9). The ivory module was over-represented for a large number of GO terms including genes annotated as enzymes that perform oxidation–reduction as well as catalyse transferase reactions (polyphenol oxidases, dehydrogenases, and transferases; Supplementary Tables S8, S10). The detected genes in these categories could be of significance because of their predicted role in catalysing missing steps in A/S biosynthesis^12^. Finally, the light-yellow subnetwork was enriched for genes that may participate in transporter activities (Fig. 6B; Supplementary Tables S8, S9).

A further detailed examination of the red subnetwork showed that it recovered the majority of genes that participate in A/S biosynthesis (e.g., LeGPPS, C4H, LePGT1 and LeGHQH2) and regulation (e.g., LeMYB1) (Supplementary Table S9). Although three recently described A/S-specific pathway genes (LeGHQH1, LeDSH1, and LeSAT1;^11,17,18^ did not coexpress with LePGT1 in the red module, two of them, LeGHQH1 and LeSAT1, were recovered in the ivory and light-yellow subnetworks, respectively (Supplementary Tables S9 and S10). Taken together, our results suggest that those genes which are coexpressed together with pathway-specific known genes, such as LePGT1, LeGHQH1/LeGHQH2, or LeSAT1 in the subnetworks, may represent potential candidate genes involved in A/S biosynthesis.

### WGCNA recovered candidate structural genes associated with A/S biosynthesis

Considering the high recovery of known genes in our coexpression modules, we next focused on the subnetworks to pinpoint potential candidates that might be associated with A/S biosynthesis. An earlier study in *L. erythrorhizon* showed that the biosynthesis of 4-HBA may occur via the *β*-oxidation route^31^. In other plant species, genes encoding enzymes that participate in *β*-oxidative benzoic acid formation were hypothesised to be involved in 4-HBA biosynthesis^32^. All *β*-oxidation 4-HBA biosynthetic genes coexpressed with LePGT1 in the red subnetwork (Fig. 6C). These include 4-coumarate ligase-like (4CLL), cinnamoyl-CoA hydratase (CHD), 3-ketoacyl-CoA thiolase (KAT), and thioesterase (TE; Fig. 6C).

Focusing on the A/S pathway, biosynthesis of GHQ from GBA may proceed via either decarboxylation alone or decarboxylation with subsequent hydroxylation^29^. Given that CYP450 enzymes have been proposed to participate in either of these steps, as well as in later steps of A/S biosynthesis^29^, we explored coexpression subnetworks for CYP450 candidate genes. Three CYP450s coexpressed with LePGT1 and one with LeSAT1. Two of these candidates were significantly upregulated in the plants of the MeJA treatment (Table 1).

**Table 1:** Candidate genes of alkannin/shikonin (A/S) biosynthesis in *Lithospermum officinale*.

Gene	Annotation	Module	log2FC*	FDR**
Leryth_000864	Acyltransferase	Ivory	1.09/1.21	2.61E−04/1.62E−03
Leryth_024167	Acyltransferase	Ivory	2.99/3.05	2.56E−21/6.79E−09
Leryth_024168	Acyltransferase	Ivory	2.91/2.96	1.24E−33/5.44E−11
Leryth_026974	Acyltransferase	Ivory	1.06/1.19	4.39E−04/2.48E−03
Leryth_001805	Neomenthol dehydrogenase	Ivory	1.88/1.91	6.59E−39/4.12E−12
Leryth_009058	Neomenthol dehydrogenase	Ivory	1.07/1.13	1.63E−07/2.64E−05
Leryth_010244	Neomenthol dehydrogenase	Ivory	2.73/2.79	1.30E−24/1.48E−09
Leryth_013998	Neomenthol dehydrogenase	Ivory	2.75/2.80	1.35E−28/3.37E−10
Leryth_015538	Polyphenol oxidase	Ivory	1.42/1.46	9.38E−10/4.26E−06
Leryth_019754	Polyphenol oxidase	Ivory	3.03/3.05	3.46E−23/4.70E−09
Leryth_027477	Polyphenol oxidase	Ivory	2.60/2.66	3.05E−24/2.21E−09
Leryth_021171	Ubiquinone biosynthesis O-methyltransferase (COQ3)	Red	1.22/1.27	1.34E−11/5.76E−07
Leryth_002195	Ubiquinone biosynthesis protein (COQ4)	Red	2.31/2.40	1.40E−12/9.42E−07
Leryth_019708	Ubiquinone biosynthesis protein (COQ4)	Red	1.03/1.08	8.26E−05/7.34E−04
Leryth_010005	Cytochrome P450	Red	0.96/1.08	8.56E−04/4.05E−03
Leryth_013232	Cytochrome P450	Light yellow	1.59/1.64	5.63E−16/4.05E−08
Leryth_013636	Cytochrome P450	Red	2.84/2.99	4.02E−07/1.57E−05
Leryth_021691	Cytochrome P450	Red	0.84/0.87	1.60E−09/2.03E−06
Leryth_013404	Berberine bridge enzyme/CBDS-like	Light yellow	1.49/1.56	1.43E−09/4.52E−06

Genes were identified by weighted gene coexpression network analysis. Expression of candidate genes in plants treated with methyl jasmonate (MeJA) is presented here with respect to control.

*log2FC = log2 fold change, values above and below the/are from DESeq2 and edgeR, respectively.

**FDR = False discovery rate, values above and below the/are from DESeq2 and edgeR, respectively.

Although the enzymes corresponding to the terminal step of A/S biosynthesis have been characterised and the underlying genes have already been identified^17,18^, the genes encoding enzymes in the intermediate steps (GHQ-3-OH to deoxy-A/S) remain ambiguous in *Lithospermum* species. Considering that cyclases, dehydrogenases, polyphenol oxidases and alcohol dehydrogenase could be involved in intermediate steps of A/S biosynthesis^12,14^, we explored coexpression network for genes encoding these enzymes. In our coexpression analysis, each of the red and light-yellow modules had one annotation related to cyclases and one of these genes was upregulated under A/S producing condition (Table 1). Furthermore, this gene showed 48% homology to the functionally characterised (tetrahydro) cannabidiolic acid synthase. Further, three polyphenol oxidases and four neomenthol dehydrogenases-like encoding genes coexpressed together with LeGHQH1 in the larger ivory subnetwork and showed a significant upregulation in response to MeJA (Table 1). Interestingly, in addition to LeSAT1, four genes encoding O-acyltransferases coexpressed with LeGHQH1 and could be potentially involved in A/S derivative biosynthesis (Table 1).

In addition to A/S pathway-specific genes, three ubiquinone pathway genes coexpressed with LePGT1 in the red subnetwork and were upregulated in response to MeJA (Table 1). Two of these genes are predicted to encode a COQ4 like ubiquinones biosynthesis protein, while the third coexpressed gene was identified as COQ3-like methyltransferase (Table 1).

### Putative regulators of A/S biosynthesis in *L. officinale*

Transcription factors (TFs) are considered the main drivers of JA-responsive SM regulation in plants^33^. Within the red subnetwork, 16 genes encoding members of MYB, ERF, JAZ, WRKY and bHLH TF families coexpressed with LePGT1 (Table 2). Based on functional annotations, four of these TFs (MYC2, MYB1, MYB4, and WRKY40) have a demonstrated role in the regulation of different SM biosynthesis, while other TFs appear to be either involved in JA-signalling (e.g., JAZs; Table 2) or regulate various biological processes (e.g., TCP4; Table 2).

**Table 2:** Putative regulators of A/S biosynthesis in *Lithospermum officinale*.

TF	Gene name	Homolog	Functional annotation	log2FC*	FDR**	References
ERF113	Leryth_015351	At5g13330	Abiotic stress	1.26/1.19	8.20E−05/1.63E−06	^34^
ILR3	Leryth_018029	At5g54680	Metal homeostasis	0.56/0.54	2.38E−06/5.05E−09	^35^
MTB3	Leryth_018651	Solyc06g083980	JA-signalling	4.1/3.94	2.65E−06/5.98E−11	^36^
bHLH25	Leryth_024227	At4g37850	Plant defence	1.05/1.02	3.57E−08/1.148E−14	^37^
bHLH37	Leryth_015611	At3g50330	Plant defence	2.16/2.01	1.50E−5/1.09E−07	^37^
**MYB4**	Leryth_011527	PhMYB4	Benzenoids biosynthesis	2.36/2.25	3.30E−06/1.50E−10	^38^
**MYB1**	Leryth_008670	LeMYB1	A/S biosynthesis	2.68/2.61	2.32E−08/5.00E−18	^20^
**MYC2**	Leryth_004691	Solyc08g076930	Glycoalkaloid biosynthesis JA-signalling	1.85/1.81	1.03E−05/5.49E−09	^39,40^
**WRKY40**	Leryth_006033	MdWRKY40	Anthocyanin biosynthesis	1.63/1.52	2.22E−04/4.77E−06	^41^
TCP4	Leryth_009997	At3g15030	JA biosynthesis and several BP	1.64/1.41	4.06E−03/9.55E−05	^42^
DF1	Leryth_017332	At1g76880	Suppress root hair growth	0.3/0.28	1.03E−02/7.22E−03	^43^
JAZ1	Leryth_002343	At1g19180	JA-signalling	2.65/2.44	1.38E−04/4.65E−07	^44^
JAZ2	Leryth_010203	At1g74950	JA-signalling	2.31/2.24	8.55E−08/2.05E−15	^44^
JAZ1	Leryth_010265	At1g19180	JA-signalling	2.07/2	2.41E−06/6.98E−11	^44^
JAZ3	Leryth_011523	At3g17860	JA-signalling	0.73/0.69	1.48E−04/9.4E−06	^44^
JAZ10	Leryth_017232	At5g13220	JA-signalling	1.5/1.39	1.21E−04/1.96E−06	^44^

Transcription factors (TFs) coexpressed with LePGT1 in the red subnetwork. TFs which have been previously demonstrated to regulate SM biosynthesis in other plant species are highlighted in bold.

*log2FC = log2 fold change, values above and below the/are from edgeR and DESeq2, respectively.

**FDR = False discovery rate, values above and below the/are from edgeR and DESeq2, respectively.

## Discussion

Phytohormones such as jasmonates (JA) and salicylic acid (SA) or their methyl derivatives have promotional effects on the production of various specialized metabolites in plants^45–47^. In Boraginaceae, alkannin/shikonin (A/S) respond uniquely to MeJA but not to SA^1,23^) as also supported by the present study. However, the mechanistic understanding of this distinct response at the transcriptional level remains largely unknown. Therefore, in the present study, we used for the first time a whole plant in vitro system to shed light on the underlying transcriptional mechanism based on comparative transcriptomics using *Lithospermum officinale* (Boraginaceae) as our model organism.

In other plant species it already has been shown that both phytohormones JA and SA are able to induce the expression of key genes of the mevalonate and phenylpropanoid pathways, two precursor pathways of the A/S biosynthesis route. For example, in *Ginkgo biloba,* JA and SA enhanced the expression of GbHMGR with a significant increase in the production of total terpene lactones^48^. In *Salvia miltiorrhiza*, both phytohormones increased the levels of key genes involved in tanshinone biosynthesis, a metabolite that is also derived from the mevalonate pathway^49,50^. Similar to the mevalonate pathway genes, SA or its methyl derivative (MeSA) enhanced the expression of key genes of the phenylpropanoid pathway in various species^47,51,52^. From these studies, we expected that SA induced the expression of key genes of A/S precursor pathways and that non-induction could be attributed to low expression of the main genes either at GBA or at GHQ-3-OH level since metabolic pathways branched at this point to other pathways. However, on the contrary, our comparative transcriptome analysis identified that none of the genes of the mevalonate pathway were differentially expressed in response to SA but showed strong expression upon MeJA treatment only. Nevertheless, genes of the other precursor pathway, phenylpropanoid, were induced in a similar way by both phytohormones. These results suggest that the low expression of the mevalonate pathway genes might have contributed to a reduced supply of precursors leading to non-detectable amounts of A/S in SA treated plants. Although the importance of the mevalonate pathway for A/S biosynthesis has also been demonstrated in earlier studies, an important novelty of our results is the characterisation of so far unidentified regulator(s) that might already exist at precursor levels to strictly control A/S biosynthesis in response to stimuli which are activated via JA and SA signalling such as against phytopathogens and herbivores.

While defence responses are crucial for plants to survive, defence activation may come at the cost of reduced plant growth^53^. Though well studied in model plant species, a mechanistic understanding of the trade-off between growth and defence related SM production such as shikonin is not clear in Boraginaceae species. In our study, both phytohormones led to a downregulation of biological processes involved in cell wall development (e.g., cell wall biogenesis, cell wall organisation). These biological processes contained xyloglucan endotransglycosylase encoding genes that have been associated with cell wall loosening and expansion^54^. Especially, the effect of MeJA on plant development was confirmed by the stunted plant growth, short roots and reduced foliar biomass. In contrast, no such phenotype was evident in SA treated plants. This difference could be attributed to the expression of additional genes unique to MeJA treatment. For example, three genes annotated as PELPK1 were downregulated in MeJA treated plants. In *A. thaliana*, PELPK1 silencing led to a reduced growth^55^. In addition, we observed that the gibberellic acid (GA) metabolism and signalling was altered by MeJA (Supplementary Table S12) through an upregulation of GA2ox8 and DDF2/DREB1F, known to participate in the production of the inactive form of GA^56–58^. In addition, GID1, involved in GA perception, and GA3OX, involved in the biosynthesis of its active forms^59,60^ were downregulated (Supplementary Table S12). We further observed that a repressor of the GA signalling pathway (DELLA2) and GA responsive bHLH159, that negatively regulate cell elongation in *A. thaliana*^61^, were upregulated in MeJA treated plants. These results suggest that a hormonal crosstalk between JA and GA might have led to the observed reduced growth of *L. officinale* as reported from model plants species^62–64^. Interestingly, GA negatively regulates A/S biosynthesis^1,65^ further suggesting crosstalk between these two phytohormones during A/S production.

Coexpression network analysis following the WGCNA methodology is a powerful approach to discover biomarkers. Numerous studies have used this approach to reveal candidate genes involved in secondary metabolism in plants^66–68^. We used this approach to identify candidate genes connected to A/S biosynthesis in *L. officinale*. By correlating the global network with the phytohormonal treatments (MeJA and SA), three subnetworks that showed a strong association with the A/S producing condition (MeJA) were identified. These subnetworks not only highlighted a strong connection of A/S pathway genes with known mevalonate and phenylpropanoid genes but also identified genes encoding enzymes that may be candidates for the precursor 4-HBA and A/S biosynthesis. For example, the *β*-oxidative benzenoid pathway genes (CHD, KAT and TE) coexpressed together with LePGT1. Homologs of one or more of these genes were characterized in *Petunia hybrida* and *Arabidopsis thaliana* to participate in benzoic acid biosynthesis and were further proposed to catalyse the formation of 4-HBA^32,69^. The first step of benzoic acid biosynthesis via the *β*-oxidative route initiates with the activation of cinnamic acid to cinnamoyl-CoA by cinnamate-CoA ligase^70^. However, we found that all three copies of cinnamate-CoA ligase were downregulated in MeJA treated plants (Fig. 6C). This together with the coexpression of 4-CLL, that catalyses the first step of the *β*-oxidative pathway of 4-HBA^71^, with remaining *β*-oxidative pathway genes might suggest an important role of 4-CLL, CHD, KAT and TE in 4-HBA biosynthesis in *L. officinale*. Since the *β*-oxidative pathway takes place in the peroxisome, candidate genes are thus expected to possess conserved peroxisomal targeting sequences. Indeed, sequence analysis and alignments with characterised genes showed that all candidates contain a typical peroxisomal targeting sequence I, except for KAT, which is characterised by a peroxisomal targeting sequence II conserved motif (Supplementary Figs. 3–6). Furthermore, one of the peroxisomal 4-CLL (Leryth_018919) was downregulated in LeGPPS RNAi hairy root lines of *L. erythrorhizon*^29^*.* Considering that the *β*-oxidative pathway contributes to 4-HBA biosynthesis^32^, the genes uncovered here thus represent strong candidates.

For the A/S pathway, among others, four CYP450 homologs were identified which are predicted to encode CYP72A, CYP707A2, CYP92C6 and CYP76B6-like proteins. Members of these CYP450 families are shown to catalyse the different reactions of terpenoid-derived SMs^72–74^, thus making these genes potential candidates to be involved in the core A/S pathway. Among non-CYP enzymes, genes encoding cannabidiolic acid synthase, dehydrogenases, and polyphenol oxidases were found to be coexpressed with LeGHQH1. Cannabidiolic acid synthase-like enzymes are proposed to perform cyclization of GHQ-3-OH, where the resultant intermediate might undergo oxidation by dehydrogenases and further oxidised by polyphenol oxidases to yield intermediates which are further converted to deoxy-A/S by unidentified enzymes^12^. Although Takanashi et al.^12^ recently pointed to these enzymes in the A/S pathway in *L. erythrorhizon*, that study was limited by incomplete or absent sequence information^17^. Furthermore, the identity of the gene encoding polyphenol oxidase remains obscure since none of the transcripts corresponding to this enzyme had considerable expression in the study of Takanashi et al.^12^. Moreover, our coexpression analysis recovered four additional O-acyltransferases genes that could be involved in stereospecific biosynthesis of A/S derivatives. One of these O-acyltransferases (Leryth_000864) belongs to the same four-member clade of A/S O-acyltransferases as that of LeSAT1 and LeAAT1^75^. Intriguingly, LeAAT1, which is involved in alkannin derivative biosynthesis, had a significant negative expression in response to MeJA. In contrast, we observed an almost equal amount of total A/S in plants treated with MeJA at six wpi (Supplementary Fig. 7). These contrasting results strongly suggest that other O-acyltransferases might potentially be involved in alkannin derivative biosynthesis. Taken together, the genes discovered in our study not only complement the earlier proteomic study of Takanashi et al*.*^12^ but unravel additional candidates of the A/S biosynthetic pathway.

Ubiquinones are important molecules that act as electron carriers in plants^71^. Both, A/S and ubiquinone biosynthesis share several similarities: First, both depend on the phenylpropanoid- and mevalonate-derived 4-HBA and the prenyl side chain precursors, respectively^71,76^). Second, the gene encoding poly prenyltransferase (PPT), an enzyme that catalyses the condensation of 4-HBA and prenyl side chain to yield prenylated 4-HBA for ubiquinone biosynthesis, shares the same evolutionary origin to that of LePGT1 and LePGT2 of the A/S pathway^76^. This is because a gene duplication event of PPT of the ubiquinone pathway has given rise to LePGT1 and LePGT2^76^. Third, A/S and ubiquinone pathways require similar ring modification steps for the biosynthesis of their end products^76^. Finally, both pathways require the decarboxylation and hydroxylation of the prenylated 4-HBA^76^. Based on these astonishing similarities, a very recent study proposed that there might be a tight evolutionary link between A/S and ubiquinone biosynthetic pathways, and that paralogs of the ubiquinone pathway genes might serve important functions in A/S biosynthesis^29^. In line with this, we identified three genes of the ubiquinone pathway that were coexpressed together with the LePGT1. One of the COQ4 encoding gene (Leryth_019708) was unique to our study, while the other two genes (Leryth_002195 and Leryth_021171) have already been considered as A/S pathway candidates by Suttiyut et al.^29^.

**Figure 7:**
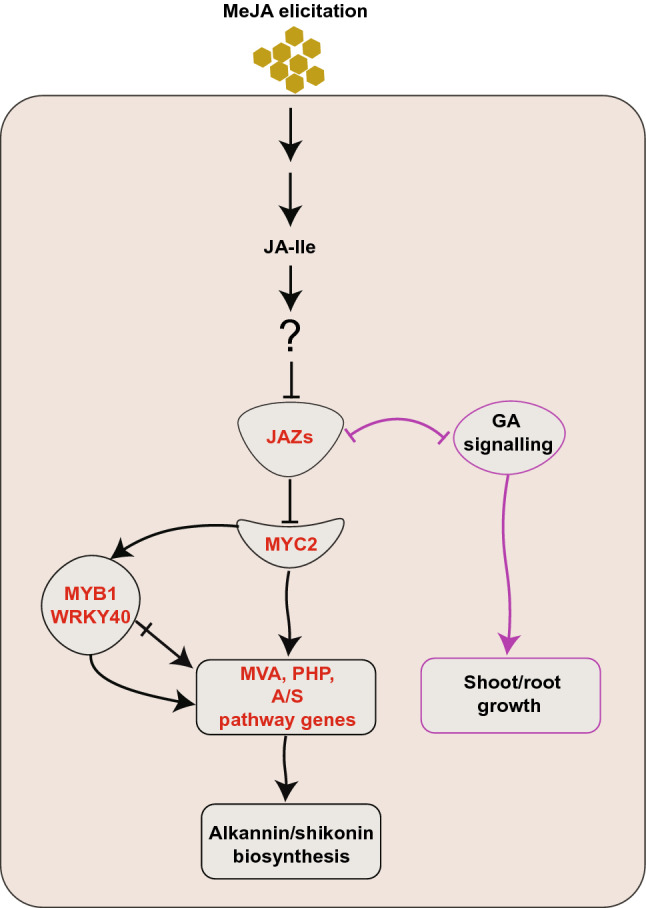
Hypothetical model of MeJA mediated A/S biosynthesis and growth reduction in *L. officinale*. MeJA elicitation leads to increased biosynthesis of JA-Ile which might be perceived by unidentified COI1 receptors leading to degradation of JAZs and relieving repression of MYC2. MYC2 might then directly upregulate the expression of precursors (mevalonate and/or phenylpropanoid) and/or A/S pathway genes leading to enhanced A/S biosynthesis. LeMYB1 and WRKY40 might directly increase the expression of pathway genes (crossed arrow) or interact with MYC2 to regulate A/S biosynthesis. Cross talk of MeJA-mediated JAZs with GA signalling might have led to the reduced growth of *L. officinale*. Blunt arrows represent inhibitory effects while complete arrows depict promoting effects. Red letters show genes that coexpressed in the red subnetwork.

Although MeJA has long been known to induce A/S biosynthesis^22^, the underlying TF, except LeMYB1^20,77^, that regulate this pathway in response to MeJA have not been fully elucidated yet. Using the red coexpression model built in the present study, we uncovered a module of TF comprising JAZs and their potential interacting partner MYC2 that might be controlling A/S biosynthesis and perhaps also other JA-mediated biological processes (Fig. 7). Both, MYC2 and JAZs (JAZ1, JAZ2 and JAZ10) were highly induced upon MeJA treatment (Table 2). This interpretation, that the MYC2-JAZ interaction and an unidentified jasmonate receptor might work as a module to control A/S biosynthesis in *L. officinale* is in line with previous studies in model plant species where JAZ-MYC2 together with the JA-Ile receptor COI1 has been shown to regulate SMs biosynthesis (reviewed in^33^). For example, increased biosynthesis of nicotine in response to JA was shown to operate in a JAZ-COI1-MYC2 dependent way^33,78^. Furthermore, JA-induced anthocyanin production was also demonstrated to operate in a similar fashion in *A. thaliana*^79^. Besides this, we also found additional uncharacterized novel transcription factors (TFs) within the red coexpression network. One of these TFs, WRKY40, has been recently demonstrated to regulate wound-dependent anthocyanin biosynthesis in *Malus domestica* through its interaction with MdMYB1^41^ and was upregulated in response to MeJA treatment in *L. officinale* roots (Table 2). It can be speculated that a similar mechanism might also operate in A/S regulation since previously characterised LeMYB1 showed enhanced expression in response to MeJA (Table 2) and coexpressed in the same red subnetwork. Alternatively, it is also possible that WRKY works in a MYC2-dependent manner as has been observed for the case of artemisinin (WRKY1) biosynthesis^80^. Regardless of how coexpressed transcription factors might be interacting, these results not only provide potential mechanistic insights into A/S regulation but also unravel novel regulatory targets for future functional studies.

## Conclusions

In conclusion, the present study enhances our understanding of A/S biosynthesis in response to the phytohormones MeJA and SA in *L. officinale*. Our data show that the non-induction of A/S upon SA treatment could be due to a lower expression of key genes encoding enzymes of the mevalonate pathway, which in turn might reduce the supply of precursors for the downstream A/S biosynthesis. Furthermore, our data suggest a hormonal cross talk between jasmonate and gibberellic acid, which might be responsible for the observed reduced growth of MeJA-treated *L. officinale* plants. A coexpression network analysis further reveals candidates likely to be involved in biochemical steps of A/S biosynthesis and regulation of A/S production. Considering the increasing interest in A/S biosynthesis, the newly generated transcriptomic data sets and coexpression models might represent important functional genomic resources aiding in deciphering the A/S pathway.

## Materials and methods

### Plant material and experimental setup

All procedures were conducted in accordance to the institutional, national, and international guidelines and legislation. The in vitro shoot cuttings of *L. officinale* (clone 16) were grown and maintained on a modified Murashagi and Skoog culture medium (MS^mod^; Supplementary Table 11;^26^). An outline of the experimental setup and in vitro system is depicted in Fig. 2. Briefly, a modified medium was used during the trial (MSR^mod^; Supplementary Table 11) and was supplemented with 10 µM MeJA, 5 µM SA, or 0.002% DMSO as a control (CO) treatment. Kumar et al.^24^ showed that A/S production decreased substantially even at low SA concentrations (10 µM). In contrast, Yazaki et al.^23^ described that SA had no noticeable effect on shikonin production in the range of 0–1000 µM. Hence, we decided to use 5 µM SA to minimise potential inhibition. Three shoot cuttings (2–3 cm in length) of 2 weeks old, rooted plantlets were transferred into each glass jar containing ~ 100 mL of MSR^mod^ medium supplemented with either treatment.

To keep the roots in the dark, as light inhibits the production of A/S ^1^, we covered the medium with sterilised sand, 1–2 cm high (oven sterilised), and wrapped the periphery of the glass jars with aluminium foil, such that the foliar part was exposed to light, while the roots remained in the dark (Fig. 2). The glass jars containing the plantlets were maintained at 20 °C under a light regime of 16 h light/8 h dark, with a light intensity of 50 µmol m^−2^ s^−1^. All jars were randomly placed and moved twice a week to avoid any positional bias. Treated plants were harvested four, six and eight wpi. At harvest, the root and shoot of every plant were separated. The root was cleaned under running tap water, quickly dried with towel paper, and flash-frozen in liquid N_2_. The root samples were further stored at − 80 °C. The shoot length and fresh weight of the shoot system were measured, and then dried for 72 h in an oven at 70 °C for shoot dry weight estimation.

Each treatment (n = 3) and time point (n = 3) consisted of three to five glass jars, each containing three individual plants. Per jar, we used two plants for metabolic analysis and one for transcriptomic analysis, summing up to 39 samples and 35 samples, respectively.

### Extraction of A/S and phytohormones

For metabolite analysis, roots of two plants from each glass jar were pooled and ground to a fine powder in liquid nitrogen. For the analysis of A/S, 35 mg of lyophilized powdered root was extracted in 1.5 mL of methanol in an ultrasonic bath at 10% power for 3 h (Bandelin Sonorex Digital 10P, Berlin, Germany) followed by centrifugation for 10 min at 12,500 rpm (Hermle Z 216 MK, Wehingen, Germany). The supernatant was collected and then filtered with 0.22 μm syringe filters. For phytohormonal analysis, ~ 10 mg of the lyophilized root powder was dissolved in 1 mL of methanol containing the mix of the following chemicals as internal standards: D6-JA (40 ng, HPC Standards GmbH, Germany), D4-SA (40 ng, Santa Cruz Biotechnology, USA), D6-abscisic acid (40 ng, Toronto Research Chemicals, Toronto, Canada), D6-JA-isoleucine conjugate (8 ng, HPC Standards GmbH, Germany), and D5-indole-3-acetic acid (OlChemIm s.r.o., Olomouc, Czech Republic). After mixing for 30 min at room temperature, the samples were centrifuged at 4 °C and 14,000 rpm for 20 min. The supernatant was used for LC–MS/MS analysis. Sample preparation for chiral analysis of alkannin and shikonin are described in Supplementary Methods.

### Quantification of A/S and phytohormones

A/S quantification was performed as described previously^26^. Briefly, A/S derivatives were quantified using HPLC coupled with DAD at the Laboratory of Organic Chemistry, Department of Chemical Engineering, Aristotle University of Thessaloniki, Greece. For A/S derivatives quantification, a wavelength of 520 nm was chosen. The following standards, purified by column chromatography, were used for metabolites identification and quantitation: alkannin (Ikeda, Japan), shikonin (Ichimaru, Japan), acetylshikonin (ABCR GmbH, Germany), deoxyshikonin (TCI, Belgium), *β, β*-dimethylacrylshikonin (ABCR GmbH, Germany), and isovalerylshikonin (TCI, Belgium).

Analyses were performed on an ECOM analytical HPLC instrument, model ECS05 (Prague, Czech Republic), utilising a Fortis SpeedCore C18 column (Cheshire, United Kingdom). The mobile phase consisted of ultrapure water (A) and acetonitrile (B). Each run lasted 13 min with a flow rate of 1 mL/min, and the samples were run in a randomised sequence to avoid bias. Data was processed with the software Clarity (DataApex, Prague, Czech Republic). Elution was performed using the following solvent gradient: 0 min 30A/70B, 8 min 100B, 13 min 100B. Prior to the next injection, the column was equilibrated for 5 min with the initial solvent composition. The column temperature was kept at 35 °C. Chiral analysis of alkannin and shikonin are described in Supplementary Methods.

Phytohormone quantification was performed at the Department of Biochemistry, Max Planck Institute for Chemical Ecology, Jena, Germany by LC–MS/MS as described in Heyer et al*.*^81^ on an Agilent 1260 series HPLC system (Agilent Technologies) with the modification that a tandem mass spectrometer QTRAP 6500 (SCIEX, Darmstadt, Germany) was used. Chromatographic separation was achieved on a Zorbax Eclipse XDB-C18 column (50 × 4.6 mm, 1.8 µm, Agilent Technologies). Water containing 0.05% formic acid and acetonitrile were used as mobile phases A and B, respectively. The elution profile was: 0–0.5 min, 10% B; 0.5–4.0 min, 10–90% B; 4.0–4.02 min, 90–100% B; 4.02–4.5 min, 100% B and 4.51–7.0, min 10% B. Flow rate was kept at 1.1 ml/min and column temperature was maintained at 25 °C. The mass spectrometer was equipped with a Turbo spray ion source operated in negative ionisation mode. The ion spray voltage was maintained at − 4500 eV. The turbo gas temperature was set at 650 °C. Nebulizing gas was set at 60 psi, curtain gas at 40 psi, heating gas at 60 psi, and collision gas was set to “medium”. The mass spectrometer was operated in multiple reaction monitoring (MRM) mode. Further details of the instrument parameters and response factors for quantification can be found in Supplementary Table S13. Since we observed that both, the D6-labelled JA and D6-labelled JA-isoleucine standards (HPC Standards GmbH, Cunnersdorf, Germany) contained 40% of the corresponding D5-labelled compounds, the sum of the peak areas of D5- and D6-compound was used for quantification. Indole acetic acid was quantified using the same LC–MS/MS system with the same chromatographic conditions but using positive mode ionisation with an ion spray voltage at 5500 eV. Multiple reaction monitoring (MRM) was used to monitor analyte parent ion → product ion fragmentations as follows: m/z 176 → 130 (collision energy [CE] 19 V; declustering potential [DP] 31 V) for indoleacetic acid (IAA); m/z 181 → 133 + m/z 181 → 134 + m/z 181 → CE 19 V; DP 31 V) for D5-indoleacetic acid.

### RNA isolation, library preparation and mRNA sequencing

For RNA isolation, ~ 40 mg root tissue of each individual plant was grinded to a fine powder in liquid nitrogen and total RNA was extracted using the RNeasy Plant Mini Kit (Qiagen, Hilden, Germany). Isolation was done according to the manufacturer’s protocol with the following modifications: (i) heating of samples at 56 °C for 3 min after addition of RLT buffer, and (ii) DNAse treatment using RNase-Free DNase Set (Qiagen, Hilden, Germany) before the washing step with buffer RW1. For DNAse treatment, we followed the instructions as described in the manufacturer’s protocol. The mRNA library preparations and sequencing were outsourced to the Next Generation Sequencing Facility of the Vienna BioCenter Core Facilities (VBCF), Austria. The mRNA libraries (n = 35) were prepared using a polyA capture method (NEB, poly-A) and were sequenced as paired-end (PE 125 bp) on the Illumina HiSeq 2500 platform on eight lanes.

### RNA seq data analysis

The quality of the raw reads was assessed using FastQC v0.11.5^82^, and raw reads were further preprocessed to remove adapters and low-quality reads (Q < 20) using BBDuk v37.68^83^ with default parameters. In addition, reads shorter than 50 bp were discarded. Lacking a *Lithospermum officinale* genome, instead of performing a de novo transcriptomics approach, the resulting high-quality reads were mapped to the genome of the closely related *Lithospermum erythrorhizon* v1.0^76^ with HISAT2 v2.1.1^84^ using default parameters by specifying the strandedness (*–RF*). Mapping quality was inspected using Qualimap v2.2.1^85^. Finally, abundance estimation at the gene level was performed using featureCounts^86^ in paired-end and strand-specific mode in R studio.

The analysis of differential expression of genes was performed with the R package DESeq2 v 1.3.0^87^ and edgeR v3.32.1^88^ by setting control samples as reference levels. Initially, we assessed the effect of each treatment and time point by performing a PCA using the plotPCA function of DESeq2 on the variance stabilizing transformed count data. Since we did not observe major differences among sampling time points, we performed analysis by controlling for the time factor using a generalized linear model ~ Time + Treatment and extracted coefficients of interest. Genes were considered significant if |logFC > 1| and the false discovery rate (FDR) was < 0.05. To improve the accuracy, only differentially expressed genes (DEG) commonly identified by both methods were considered for further analysis. Overrepresentation of gene ontology (GO) terms associated with DEGs was assessed using one-sided Fisher’s exact test implemented in R package topGO v2.42^89^. GO terms were considered overrepresented if *p* < 0.01 and were visualized using the R package GOplot^90^.

### Weighted gene coexpression network analysis

A gene coexpression network analysis was performed using the R package WGCNA v1.70-3^30^. As input data, vst count data (see above) were used, where genes with low expression values (normalized counts < 5 in at least 85% of samples) were filtered out. To increase specificity, four samples showing outlier position in the distance-based tree were removed from this analysis (106,273, 106,283, 106,323 and 106,315; Supplementary Figure S8). For the construction of coexpression modules, a soft threshold power (*β*) of 15 was chosen as this is the lowest power for which the scale-free topology index reached 0.9. Modules were merged using mergeCloseModules using a cutHeight of 0.25. To identify which modules might be associated with the treatments (MeJA or SA), we estimated the correlation of each treatment with the module eigengene (i.e., expression of genes summarised as the first principal component). We expected that the modules which are positively and significantly correlated with the MeJA treatment would harbour genes related to A/S metabolism. The identified modules were then used to estimate the gene significance and module membership of each gene to the specific treatment.

## Supplementary Information

Supplementary Figures.

Supplementary Information.

Supplementary Tables.

## Data Availability

Raw sequencing reads have been deposited in NCBI under the accession number PRJNA792892 and can be accessed via https://www.ncbi.nlm.nih.gov/bioproject/PRJNA792892.
