# Supplementary material for: Dynamics of alkannin/shikonin biosynthesis in response to jasmonate and salicylic acid in Lithospermum officinale
Source: Sci Rep. 2022 Oct 12;12:17093. doi: 10.1038/s41598-022-21322-0 (PMC9554848; doi:10.1038/s41598-022-21322-0)
Supplement: Supplementary file 1 — Supplementary Figures. [file 41598_2022_21322_MOESM1_ESM.pdf]

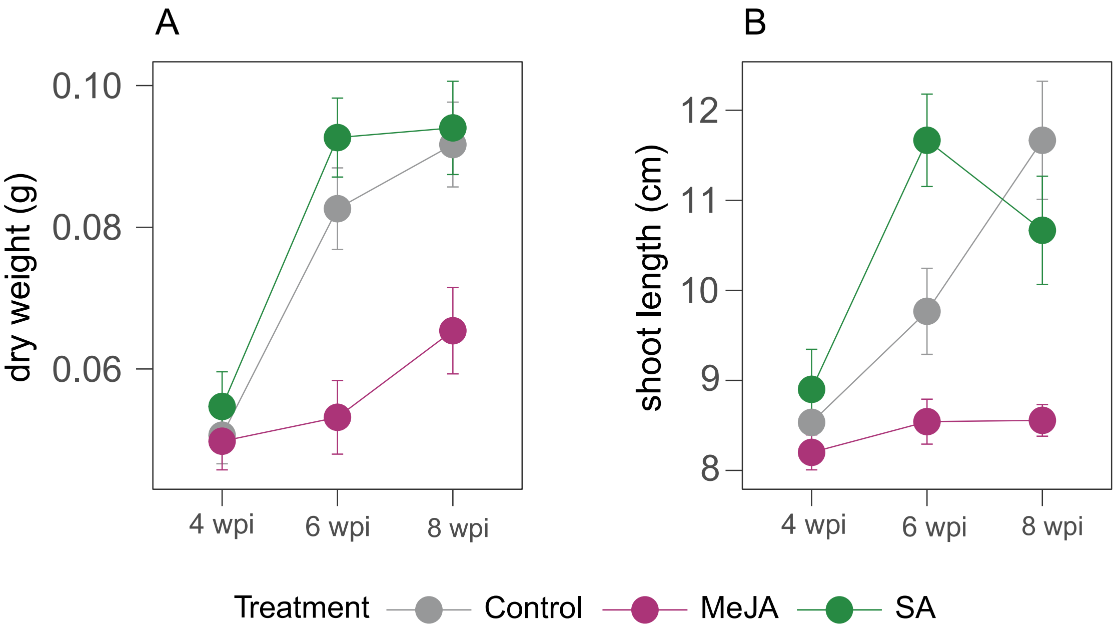

Supplemental Figure S1: Comparison of growth phenotypes between the treatments on three time points. A) Diagram of the evolution of the shoot dry weight of the plants from the different treatments at 4, 6 and 8 wpi. **B)** Diagram of the evolution of the shoot length of the plants from the different treatments at 4, 6 and 8 wpi.

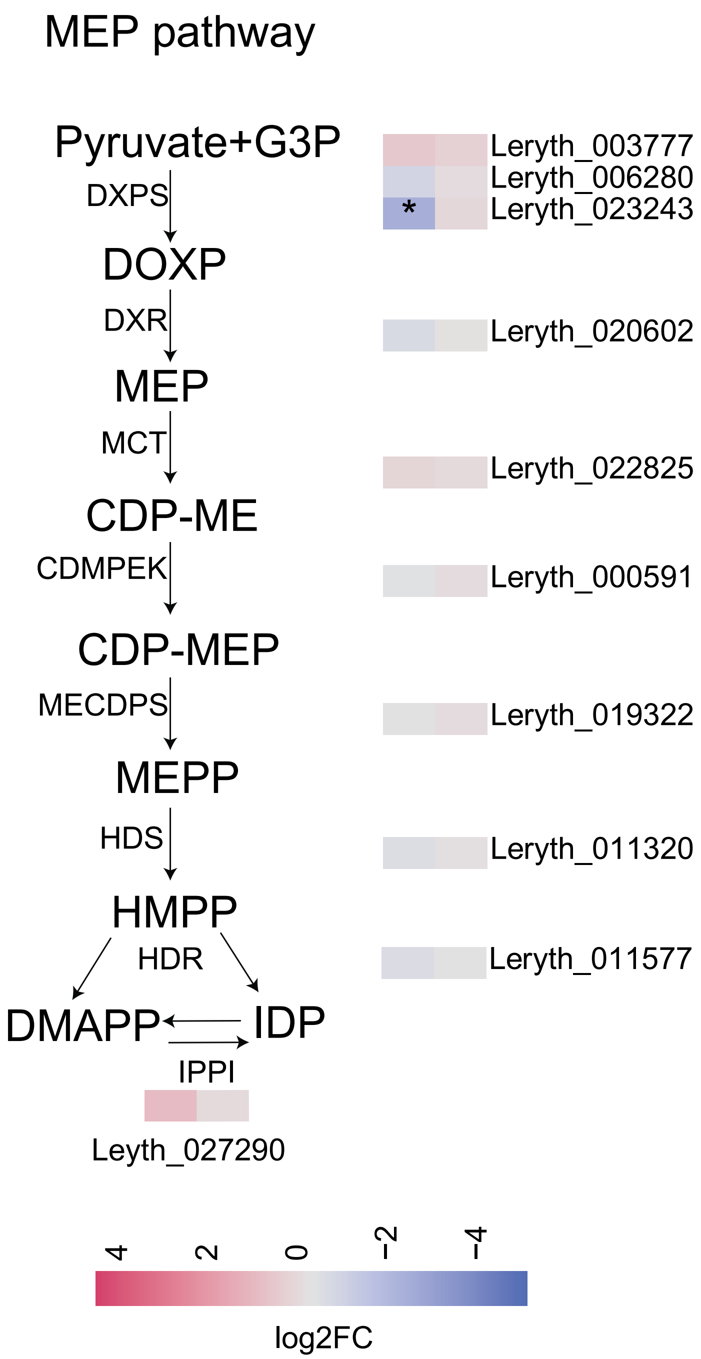

Supplemental Figure S2. Effect of methyl jasmonate (MeJA) and salicylic acid (SA) on the expression of genes involved in the plastidal methylerythritol phosphate (MEP) pathway. The mean log2 fold-change in expression level of MeJA and SA treated roots as compared to control in the methylerythritol phosphate (MEP) pathway. * Denotes differentially expressed gene at log2fold > |1| and FDR <0.05. Abbreviations: G3P, D-glyceraldehyde 3-phosphate, DXPS, 1-deoxy-D-xylulose-5-phosphate synthase; DOXP, 1-deoxy-D-xylulose 5-phosphate, DXR, 1-deoxy-D-xylulose-5-phosphate reductoisomerase; MEP, 2-methylerylthritol 4-phosphate, MCT, 2-*C*-methyl-D-erythritol 4-phosphate cytidylyltransferase; CDP-ME, 4-Diphosphocytidyl-2-C-methylerythritol; CDPMEK, 4-(cytidine 5′-diphospho)-2-*C*-methyl-D-erythritol 2-phosphokinase; CDP-MEP, 4-diphosphocytidyl-2-C-methylerythritol 2-phosphate, MECDPS, 2-*C*-methyl-D-erythritol 2,4-cyclodiphosphate synthase;

MEPP, methylerythritol cyclodiphosphate; HDS, 1-hydroxy-2-methyl-2-butenyl-diphosphate synthase; HMPP, (E)-1-hydroxy-2-methylbut-2-enyl 4-diphosphate; HDR, 1-hydroxy-2-methyl-2-(*E*)-butenyl-4-diphosphate reductase; DMAPP, Dimethylallyl pyrophosphate; IDP, isopentenyl diphosphate; IPPI, isopentenyl pyrophosphate isomerase

```
                                   10        20        30        40
                          ----:----|----:----|----:----|----:----|----:---
Consensus                 MAEILXANX-SQTXTXXSSQXYPXWYSPKTGIYHSKHTSXXLPTEPFX
At4CLL6                   MAATHLHIP-PNPKTQTSHQNPPFWFSSKTGIYTSKFPSLHLPVDPNL
Leryth_018919-t1          MGEILKANE-SQTITTPSSQAYPSWYSPKTGIYHSKHTSMNLPTEPFA
Leryth_018919_complete    MGENLKANE-SQTITTPSSQAYPSWYSPKTGIYHSKHTSMNLPTEPFA
Leryth_004695-t1          ------------------------------------------------
Leryth_004695_Complete    MAEILRAFPQTSSKTFSDEASYPYWYSPETGIYQSKHTPVALPTEPFV
Leryth_000173-t1_Complete MAELFRA----DAQTFSEDRSYPYWYSPETGIYHSKHAKVALPIEPFV
Leryth_000173-t1          ------------------------------------------------

                           50        60        70        80        90
                          -|----:----|----:----|----:----|----:----|----:-
Consensus                 DAVSYIFSHKHXGXTALIDSSTGVSIPYSQLYXSVKAXASGLHH-MGV
At4CLL6                   DAVSALFSHKHHGDTALIDSLTGFSISHTELQIMVQSMAAGIYHVLGV
Leryth_018919-t1          DVVSYIFSQKHEGENALVDSSTGVSIPYSQLYPSVKAVASGLHH-MGV
Leryth_018919_complete    DVVSYIFSQKHEGENALVDSSTGVSIPYSQLYPSVKAVASGLHH-MGV
Leryth_004695-t1          ------------------------------------------------
Leryth_004695_Complete    DAVSYIFSHKHDGVTALIDSSSGVSIPYSQLYSSVKAIASGLHQ-MGV
Leryth_000173-t1_Complete DAVSYIFSHKHDGVTALIDSTTGVSIPYSQLYSSVKAIASGLHQ-MGV
Leryth_000173-t1          ---------------------------------------------MGV

                             100       110       120       130       140
                          ---|----:----|----:----|----:----|----:----|----
Consensus                 KQGDVVLISLPNSIIFPIIFLGVLSJGAIVAPMNPVSNPLEIKKQTKD
At4CLL6                   RQGDVVSLVLPNSVYFPMIFLSLISLGAIVTTMNPSSSLGEIKKQVSE
Leryth_018919-t1          KQGDVVLILLPNSIIFPIIFLGVLSLGATIAPMNPVSNPLEIKKQTKD
Leryth_018919_complete    KQGDVVLILLPNSIIFPIIFLGVLSLGATIAPMNPVSNPLEIKKQTKD
Leryth_004695-t1          ------------------------------------------------
Leryth_004695_Complete    QQGDVVLISLPNSIIFPIIFLGVLSIGAIVAPMNPVSNPLEIKKQTKD
Leryth_000173-t1_Complete KQGDVVLISLPNSIIFPIIFLGVLSIGAIVAPMNPVSNPLEIKKQTKD
Leryth_000173-t1          KQGDVVLISLPNSIIFPIIFLGVLSIGAIVAPMNPVSNPLEIKKQTKD

                               150       160       170       180       190
                          :----|----:----|----:----|----:----|----:----|--
Consensus                 CKVKXAFTTPEKVXQXSXA-LNLPAIPVSENITPGSI------FHKII
At4CLL6                   CSVGLAFTSTENVEKLSS--LGVSVISVSESYDFDSIRIENPKFYSIM
Leryth_018919-t1          CEVKLAFTTPEKVEQLKSAGLQFPTIPVQENIIPGSI------FHTII
Leryth_018919_complete    CKVKLAFTTPEKVEQLKSAGLQFPTIPVQENIIPGSI------FHTII
Leryth_004695-t1          ------------------------------------------------
Leryth_004695_Complete    CKVKFAFTTPEKVDQVSKA-LNLPAIPVSENITPGSI------FHKII
Leryth_000173-t1_Complete CKVKFAFTTPEKVDQVSKA-LNLQAIPVSENITPGSI------FHKII
Leryth_000173-t1          CKVKFAFTTPEKVDQVSKA-LNLQAIPVSENITPGSI------FHKII

                                 200       210       220       230       240
                          --:----|----:----|----:----|----:----|----:----|
Consensus                 SSDPNXAPKPRINQEDTAAIMFSSGTTGACKGVVLTHXNFIXMVALFV
At4CLL6                   KESFGFVPKPLIKQDDVAAIMYSSGTTGASKGVLLTHRNLIASMELFV
Leryth_018919-t1          TSDPNFAPKPIINQEHAAAIMFSSGTTGACKGVVLTHKNFIAMVALFI
Leryth_018919_complete    TSDPNFAPKPIINQEHAAAIMFSSGTTGACKGVVLTHKNFIAMVALFI
Leryth_004695-t1          --------------------MFSSGTTGACKGVVLTHRNFISMVALFV
Leryth_004695_Complete    SSDPNLAPKPRISQEDTAAIMFSSGTTGACKGVVLTHRNFISMVALFV
Leryth_000173-t1_Complete SSDPNLAPKPRINQEDTAAIMFSSGTTGACKGVVLTHKNFISMVALFV
Leryth_000173-t1          SSDPNLAPKPRINQEDTAAIMFSSGTTGACK-----------------
```

```
                                   250       260       270       280
                          ----:----|----:----|----:----|----:----|----:---
Consensus                 RFEXSQY-EFTPSKNVYLAVLPMFHIFGLAQFVTGLLSLGTTIVVMKK
At4CLL6                   RFEASQY-EYPGSSNVYLAALPLCHIYGLSLFVMGLLSLGSTIVVMKR
Leryth_018919-t1          RFEASQYKELSPSKNVYLAVLPMFHIFGLAQFVTGLLSLGTTIIVMKK
Leryth_018919_complete    RFEASQYKELSPSKNVYLAVLPMFHIFGLAQFVTGLLSLGTTIIVMKK
Leryth_004695-t1          RFESSQY-SFTPSKNVYIAVLPMFHIFGLAQFVTGLLSLGTTVVVMKK
Leryth_004695_Complete    RFESSQY-SFTPSKNVYIAVLPMFHIFGLAQFVTGLLSLGTTIVVMKK
Leryth_000173-t1_Complete RFESSQY-PFTPSKNVYLAVLPMFHIFGLAQFVTGLLSLGTTIVVMKK
Leryth_000173-t1          -----------------------------AQFVTGLLSLGTTIVVMKK

                           290       300       310       320       330
                          -|----:----|----:----|----:----|----:----|----:-
Consensus                 FDPDEMVKAIEKFGVTHFPVVPPLMTALTKKAKSXDVKXGNFLRSLKQ
At4CLL6                   FDASDVVNVIERFKITHFPVVPPMLMALTKKAKGVC---GEVFKSLKQ
Leryth_018919-t1          FDPEEMIRAIEKFRVTHFPVVPPLMNALTKKARTLP-NSSEILKSLKQ
Leryth_018919_complete    FDPEEMIRAIEKFRVTHFPVVPPLMNALTKKARTLP-NSSEILKSLKQ
Leryth_004695-t1          FNPDEMVKAIAKYGVTHFPVVPPLMTALTKKAKSMDVKKGNFLRSLKQ
Leryth_004695_Complete    FNPDEMVKAIAKYGVTHFPVVPPLITALTKKAKSMDVKKGNFLRSLKQ
Leryth_000173-t1_Complete FHPDEMVKAISRFGVTHFPVVPPLMTALTKKAKSEDVKNANFLRSLKQ
Leryth_000173-t1          FHPDEMVKAISRFGVTHFPVVPPLMTALTKKAKSEDVKNANFLRSLKQ

                             340       350       360       370       380
                          ---|----:----|----:----|----:----|----:----|----
Consensus                 VSSGAAPLTTKLIQEFVQALPHVDFIQGYGMTETTAVATRGYNTKELK
At4CLL6                   VSSGAAPLSRKFIEDFLQTLPHVDLIQGYGMTESTAVGTRGFNSEKLS
Leryth_018919-t1          VSSGAAPLTTKAIQEFVQALPHVDFIQGYGMTETTAVATRGFNTNELK
Leryth_018919_complete    VSSGAAPLTTKAIQEFVQALPHVDFIQGYGMTETTAVATRGFNTNELK
Leryth_004695-t1          VSSGAAPLTTKLIQELVQALPHVDFIQGYGMTETTAVATRGYNTKDLK
Leryth_004695_Complete    VSSGAAPLTTKLIQELVQALPHVDFIQGYGMTETTAVATRGYNTKDLK
Leryth_000173-t1_Complete VSSGAAPLTTKLIQEFVQALPHVDFIQGYGMTETTAVATRGYNTKELK
Leryth_000173-t1          VSSGAAPLTTKLIQEFVQALPHVDFIQGYGMTETTAVATRGYNTEELK

                               390       400       410       420       430
                          :----|----:----|----:----|----:----|----:----|--
Consensus                 NYTSVGLLAPNSEAKVVDFVSGAXMPPGGVGELWLRSPGVMKGYLNNI
At4CLL6                   RYSSVGLLAPNMQAKVVDWSSGSFLPPGNRGELWIQGPGVMKGYLNNP
Leryth_018919-t1          NYTSVGLLAPNSEAKVVDWVSGESMPPGGVGELWLRSPGVMKCYLKNP
Leryth_018919_complete    NYTSVGLLAPNSEAKVVDWVSGESMPPGGVGELWLRSPGVMKCYLKNP
Leryth_004695-t1          NYTSVGLLAPNSEAKVVDFVSGATMPPGGVGELWLRSPGVMNGYLNNI
Leryth_004695_Complete    NYTSVGLLAPNSEAKVVDFVSGATMPPGGVGEVWLRSPGVMNGYLNNI
Leryth_000173-t1_Complete NYTSVGLLAPNSRAKVIDFVSGAAVPPGGVGELWLRSPGVMKGYLNNI
Leryth_000173-t1          NYTSVGLLAPNSQAKVIDFVSGAAVPPGGVGELWLRSPGVMKGYLNNI

                                 440       450       460       470       480
                          --:----|----:----|----:----|----:----|----:----|
Consensus                 KETMSTVDTEGWLHTGDIVYFDQDGYLYVIDRLKEVIKYKGFQISPAD
At4CLL6                   KATQMSIVEDSWLRTGDIAYFDEDGYLFIVDRIKEIIKYKGFQIAPAD
Leryth_018919-t1          EETINNVDKDGWLHTGDIVYFDQDGYLYVI---RKVIK----------
Leryth_018919_complete    EETINNVDKDGWLHTGDIVYFDQDGYLYVIDRLKEVIKYKGFQISPAD
Leryth_004695-t1          KETMSTVDTEGWLHTGDIVYFDQDGYLYVIDRLKEVIKYKGFQISPAD
Leryth_004695_Complete    KETMSTVDTEGWLHTGDIVYFDQDGYLYVIDRLKEVIKYKGFQISPAD
Leryth_000173-t1_Complete QETMSTVDTEGWLHTGDIVYFDQEGYLYIIDRLKEVIKYKGFQISPAD
Leryth_000173-t1          KETMSTVDTEGWLHTGDIVYFDQEGYLYIIDRLKEVIKYKGFQISPAD
```

```
                                       490       500       510       520
                          ----:----|----:----|----:----|----:----|----:---
Consensus                 LESVLMSHPEVADAAVTAVTDEEAGEIPVAFVVRKKGSELSEESLMDF
At4CLL6                   LEAVLVSHPLIIDAAVTAAPNEECGEIPVAFVVRRQETTLSEEDVISY
Leryth_018919-t1          ------------------------------------------------
Leryth_018919_complete    LESVLMSHPEVVDAAVTAITDEEAGEIPVAFVVRKKGSKLSETSLMDF
Leryth_004695-t1          LESVLMSHPEVADAAVTAVTDEEAGEIPVAFVVRKKGSELSEESLMDF
Leryth_004695_Complete    LESVLMSHPEVADAAVTAVTDEEAGEIPVAFVVRKKGSELSEESLMDF
Leryth_000173-t1_Complete LESVLMSHPEVADAAVTAVTDEEAGEIPVAFVVRKKGSELSEESLMDF
Leryth_000173-t1          LESVLMSHPEVADAAVTAVTDEEAGEIPVAFVVRKKGSELSEESLMDF

                            530       540       550       560       570
                          -|----:----|----:----|----:----|----:----|---
Consensus                 VATKVSPYKKVRKVCFTNSVPRSAAGKIIRRELKMIL----PSRL
At4CLL6                   VASQVAPYRKVRKVVMVNSIPKSPTGKILRKELKRILTNSVSSRL
Leryth_018919-t1          ---------------------------------------------
Leryth_018919_complete    VATKVSPYKKVRKVCFTNVVPRSAAGKIIRRELKNIL----PSRL
Leryth_004695-t1          VATKVSPYKKVRKVCFTNSVPRSAAGKIIRRELKMIL----PSRL
Leryth_004695_Complete    VSTKVSPYKKVRKVCFTNSVPRSAAGKIIRRELKMIL----PSRL
Leryth_000173-t1_Complete VATKVSPYKKVRKVCFTSSVPRSAAGKIIRRELKMIL----PSRL
Leryth_000173-t1          VATKVSPYKKVRKVCFTSSVPRSAAGKIIRRELKMIL----PSRL
```

Supplemental Figure S3. Amino acid sequence comparison of putative *L. officinale* 4-coumarate ligases-like genes recovered in coexpression network (4CLL; Leryth_000173, Leryth_004695 and Leryth_018919) with the characterised 4CLL from *Arabidopsis thaliana* (At4CLL6). The amino acid alignments were computed using the MUSCLE algorithm implemented in Geneious. The pink highlighted sequence is the peroxisomal targeting sequence I (PTS I), predicted by PSORT (https://www.psort.org/).

```
                               10         20         30         40
                       ----:----|----:----|----:----|----:----|----:---
Consensus              MXGSMVKPTVTMEVGXDGVAVITXINPPVNXLAIPIIEGLKEKFKEAE
Leryth_018504-t1       ----MVAPTVTMEVG---------------------IIEGLTEKFKEAE
PtCHD1                 ----MAKPHVTMEVGNDGVAVVTLINPPVNALAIPIIAGLKEKFDEAT
PtCHD2                 MEGSRTKGRTTIEVGADGVALITIINPPVNSLSFDVLYSLKDSFEQAL
PtCHD3                 MKGSRTKGTTTIEVGADGVALITLINPPVNALSSDVLNSLKDSYEQAL
PhCHD                  ----MAQVKVTMEVGTDGVAVITIFNPPVNALAIPIINALKEKWTEAT
Leryth_020735-t2       ----MVVPTVTMEVGSDGVAVITMSNPPVNSLAIPIIEGLTGKFKEAE
Leryth_020735-t1       ----MVVPTVTMEVGSDGVAVITMSNPPVNSLAIPIIEGLTGKFKEAE

                         50         60         70         80         90
                       -|----:----|----:----|----:----|----:----|----:-
Consensus              RRNDVKAIVLTGKGGRFSGGFDINVFQLVHKTGDISXXPD-VSVEXVV
Leryth_018504-t1       RRNDVKAIVLTGKGGKFSGGFDINVFQLVHKTGDISHLPD-VSVSFVN
PtCHD1                 RRNDVKALVLTGKGGRFSGGFDINVFQKVHATGDISLMPD-VSVELVV
PtCHD2                 RRDDVKAIVITGAKGKFSGGFDISSFGGVQGGKSNEPKPGFISVEILS
PtCHD3                 RRDDVKAIVITGAKGKFSGGADISSFEEVQEGKVNEPKPDFIFSEVLG
PhCHD                  IRNDVKAIVLTGNGGRFSGGFDINVFQKVHGTGDISQMPD-VSVDLVV
Leryth_020735-t2       RRSDVKAIVLTGKGGRFSGGFDINVFQLVHKTG--------------V
Leryth_020735-t1       RRSDVKAIVLTGKGGRFSGGFDINVFQLVHKTG--------------V

                          100        110        120        130        140
                       ---|----:----|----:----|----:----|----:----|----
Consensus              NTXEDCKKPXVAAIEGLALGGGLELAMACHARIAAPXAQLGLPELXLG
Leryth_018504-t1       NIVEDSKKPAVAAVEGLALGGGLELAMACHARIAAPKTQLGLPELTLG
PtCHD1                 NTIEDCKKPVVAAVEGLALGGGLELAMGCHARIAAPKTQLGLPELTLG
PtCHD2                 DTVEAAKKPSVAAIDGLALGGGLEVAMACHARISTSTAQLGLPELQLG
PtCHD3                 DTLEAAKKPSVAAIDGLALGGGLEVAMACHARISTPTAQLSLPELQLG
PhCHD                  NTMEDCKKPAVAAIEGLALGGGLELAMGCHARIAAPRAQLGLPELSLG
Leryth_020735-t2       PCKDCCTKNT---------AGSLLCESCC-------------------
Leryth_020735-t1       PCKDCCTKNT---------AGSLLCESCC-------------------

                            150        160        170        180        190
                       :----|----:----|----:----|----:----|----:----|--
Consensus              XIPGFGGTQRLPRLXGLXKAIEMMXXSKPIMSEEGEKLGLIDAVVPSX
Leryth_018504-t1       IIPGFGGTQRLPRLVGLPKAIEMMVFSKPIMSEVGEKLGLIDAVVPSG
PtCHD1                 VIPGFGGTQRLPRLLGLSKAIEIMLLSKPIMSEEGKKLGLIDAIVPSQ
PtCHD2                 LIPGFGGTQRLPRLVGISKALEMMLTSKPVKGEEAHALGLVDAVVSPN
PtCHD3                 LIPGFGGTQRLPRLVGITKALEMMLTSKPVKGEEAHALGLVDAVVSPN
PhCHD                  VMPGFGGTQRLPRLIGLSKAVEMMMTSKPIMSEEGKKLGLIDAIVPSS
Leryth_020735-t2       ----FKQTQRLPRLIGLPKAIEVMVFSKPIMSEEGEKLGLIDVVVPSK
Leryth_020735-t1       ----FKQTQRLPRLIGLPKAIEVMVFSKPIMSEEGEKLGLIDVVVPSK

                              200        210        220        230        240
                       --:----|----:----|----:----|----:----|----:----|
Consensus              ELLNVARQWALDIAEXRKPWXRSLHKTDKIGSLSEAREILKGARQQVK
Leryth_018504-t1       DLLNVARRWALDIADKHKPWIRSLHKTDKIGSISEAHNILQGARQQVK
PtCHD1                 ELLKVSRQWALDISERRKPWLRSLHRTDKIGSLSEAREVLKAARQQAK
PtCHD2                 ELVSTARQWALDILERRRPWIASLYKTEKLDSLGEAREIFKFAREQVR
PtCHD3                 ELVSTARQWALDIFECRRPWIASLYKTEKLDSLGEAREIFKFARAQAQ
PhCHD                  ELLKVSRQWALDIAERRKPWMRSLHKTDKIGSLSEAREVLKVARQQVK
Leryth_020735-t2       DLLNVARKWALDIAEKRKPWMRSLHKTDKIGSISEAHKILQGARQQVK
Leryth_020735-t1       DLLNVARKWALDIAEKRKPWMRSLHKTDKIGSISEAHKILQGARQQVK

                               250        260        270        280
                       ----:----|----:----|----:----|----:----|----:---
Consensus              KTAXNXXHHQLCIDVIEXGIVHGGYHGLLKETKXFNXLVLSDXXKGLI
```

```
Leryth_018504-t1 KTFRNLVHHQLCISVMEDGIVHGGYHGVLEETKTFNKLVLSDNAKGLI
PtCHD1           KIAPNVPQHQALLDVMEEGIIHGGYNGVLKEAKVFKELVLTETSKGLV
PtCHD2           KRAPNLTHPIVCIDVIEHGIVSGPRDGLYKELESFHELVRSDTCKSLI
PtCHD3           KRAPNLLHPIVCINVVEHGIVSGPRAGLYKEFESFQELVRSDISKSLV
PhCHD            QTAKNMPQHVACIDVIEEGIIHGGYAGVLKEAKVFKDLVLSETSKGLV
Leryth_020735-t2 QTFRNMFHHQLCLDAIEAGIVHGGYHGLLEETKIFNKLVLSDNAKGLI
Leryth_020735-t1 QTFRNMFHHQLCLDAIEAGIVHGGYHGLLEETKIFNKLVLSDNAKGLI

                  290       300       310       320       330
                 -|----:----|----:----|----:----|----:----|----:-
Consensus        HIFFAQRATSKVPNVTDVGLKPRPXKKVAVIGGGLMGSGIATALILSN
Leryth_018504-t1 HIFFSQRATSKVPNVTDVGLKPNPAKKVAVIGGGLMGSGIATALVLNN
PtCHD1           HVFFAQRMTSKIPKVTDVGLKPRHIKKVAVIGGGLMGSGIATALIVSN
PtCHD2           HIFFAQRGTTKVPGITDLGLVPRRVKKVAVLGGGLMGSGIATALVLSN
PtCHD3           HIFFALRGTTKVPGITDLGFVPRLVKKVAVLGGGLMGSGIATALILSN
PhCHD            HVFFAQRATSKVPNVTDIGLKPRTVKKVAVIGGGLMGSGIATALALSN
Leryth_020735-t2 HIFFSQRATSKVPNVTDVGLKPNPAKKVAVIGGGLMGSGIATALILNN
Leryth_020735-t1 HIFFSQRATSKVPNVTDVGLKPNPAKKVAVIGGGLMGSGIATALILNN

                    340       350       360       370       380
                 ---|----:----|----:----|----:----|----:----|----
Consensus        IXVVLKEINSEYLQKGIKSVEANVRSLVXRKKLTQEKADKALSMLKGX
Leryth_018504-t1 INVVLKEINSDYLLKGIQSVEANVKSMVLRKKLTQEKANKALSLLKGV
PtCHD1           IHVVLKEINSEYLQKGTKTIEANVRSLVTRGKLTRDKADKALSMLKGA
PtCHD2           YPVILKEVNNQFLQAGIGRVRANLQSRVKKGRMTQEKFEKTMSLLKGA
PtCHD3           YPVILKEVNDQFLQAGIGRVRANLQSRVKKGKMTQEKFEKTMSLLKGS
PhCHD            TFVVLKEINSEYLQKGMKAIEANVRGLVARKKLPQDKADKALSMVKGA
Leryth_020735-t2 IYVVLKEINSEYLLKGIKSVEANVRSLVSRKKLTQEKGDKALSMLKGV
Leryth_020735-t1 IYVVLKEINSEYLLKGIKSVEANVRSLVSRKKLTQEKGDKALSMLKGV

                      390       400       410       420       430
                 :----|----:----|----:----|----:----|----:----|--
Consensus        LDYSEFKDVDMVIEAVIENVSLKQKIFSELEKICPPHCILASNTSTID
Leryth_018504-t1 LDYSEFKDVDMVIEAVIENISLKQKIFGELEKICPPHCVLASNTSTID
PtCHD1           LDYSDFKDVDMVIEAVIESVPLKQKIFSEIEKICPPHCILATNTSTID
PtCHD2           LDYESFKDVDMVIEAVIENVSLKQQIFSDLEKYCPPHCILASNTSTID
PtCHD3           LDYESFKDVDMVIEAVIENVSLKQQIFSDLEKYCPPHCILASNTSTID
PhCHD            LDYSDFKDVDMVIEAVIESVPLKQKIFSEIEKVCPPHCILASNTSTID
Leryth_020735-t2 LDYSEFADVDMVIEAVIENISLKQKIFGELEKICPPHCVLASNTSTID
Leryth_020735-t1 LDYSEFADVDMVIEAVIENISLKQKIFGELEKICPPHCVLASNTSTID

                        440       450       460       470       480
                 --:----|----:----|----:----|----:----|----:----|
Consensus        LNLVGENTXSQDRIIGAHFFSPAHVMPLLEIVRTEKTSXQVILDLMTV
Leryth_018504-t1 LNLVGEKTRSQDRIIGAHFFSPAHVMPLLEIVRTEKTSSQVILDLMTV
PtCHD1           LNLVGEKTSSQDRIIGAHFFSPAHIMPLLEIVRTEKTSAQAILDLMTV
PtCHD2           LNLIGKQTKSQDRIIGAHFFSPAHVMPLLEIVRTKQTSPQVIVDLLDV
PtCHD3           LNLIGKQTKSQDRIIGAHFFSPAHVMPLLEIVRTKQTSPQVIVDLLDV
PhCHD            LNIIGENTRSKDRIIGAHFFSPAHIMPLLEIVRTEKTSTQAILDLMAV
Leryth_020735-t2 LNLVGENTISQDRIIGAHFFSPAHVMPLLEIVRTEKTSGQVVLDLMTV
Leryth_020735-t1 LNLVGENTISQDRIIGAHFFSPAHVMPLLEIVRTEKTSGQVVLDLMTV

                          490       500       510       520
                 ----:----|----:----|----:----|----:----|----:---
Consensus        GKTIKKVPVVVGNCTGFAVNRTFFPYTQSALFLVNLGVDLFRIDQLIS
Leryth_018504-t1 GKTIKKVPVVVGNCTGFAVNRTFFPYTQSALFLVNLGVDLFRIDQLIS
PtCHD1           GKTIKKVPVVVGNCTGFAVNRAFFPYTQSALILVHLGVDVFRIDKLIS
PtCHD2           GKKIRKTPVVVGNCTGFAVNRMFFPYTQAALLLVEHGADLYQIDKVIT
```

```
PtCHD3            GKKIRKTPVVVGNCTGFAVNRMFFPYTQAAIFLVEHGVDLYQIDRVIT
PhCHD             GKAIKKVPVVVGNCTGFAVNRTFFPYSQGAHILVNLGVDAYRIDAQIT
Leryth_020735-t2  GKTIKKVPVVVGNCTGFAVNRTFFPYTQSALFLVNLGVDLFRIDQLIS
Leryth_020735-t1  GKTIKKVPVVVGNCTGFAVNRTFFPYTQSALFLVNLGVDLFRIDQLIS

                   530       540       550       560       570
                  -|----:----|----:----|----:----|----:----|----:-
Consensus         SFGLPMGPFQLQDLSGYGVAIAVGKEFAXAFPDRTLKSPLIELMIKSG
Leryth_018504-t1  NFGLPMGPFQLQDVSGYGVAIAVGKEFASAFPDRTLQSPLIELMIKSG
PtCHD1            SFGLPMGPYQLQDLSGYGVALAVEKEFANAFPDRTFQSPLIHLLVKSG
PtCHD2            KFGMPMGPFRLVDLVGFGVAIATGTQFVVNFPERTYKSMLIPLMQEDK
PtCHD3            KFGMPMGPFRLADLVGFGVAIATGMQFVENFPERTYKSMLLPLMQEDK
PhCHD             SFGLPMGPLQLQDLTGYGVAVAVGKEFGSAFSDRTFKSPLIDLLIKSG
Leryth_020735-t2  SFGLPMGPFQLQDVSGYGVAIAVGKEFATAFPDRTLRSPLIELMIKNG
Leryth_020735-t1  SFGLPMGPFQLQDVSGYGVAIAVGKEFATAFPDRTLRSPLIELMIKNG

                     580       590       600       610       620
                  ---|----:----|----:----|----:----|----:----|----
Consensus         RNGKXNGKGYYIYEKGSKPKPDPSVQSIIEESRRLTNAMPGGK-----
Leryth_018504-t1  RNGKSNGKGYYIHEKGSKPKPDPSVQSIIEESRRLTNAMVGGKVRLHP
PtCHD1            RNGKTNGKGYYIYEKGSKPRPDPSVLPIIEESRRLANIMPNGK-----
PtCHD2            RAGETTRKGFYLYDDRRKAKPDPELRKYIEKARNISGVANDPK-----
PtCHD3            RGGETTCKGFYLYDDRRKAKPDPELRKYIEKARSISGVAVDPK-----
PhCHD             RNGKNNGKGFYIYEKGSKPRPDLSVLPIIEESRRLTNIMPGGK-----
Leryth_020735-t2  RNGKSNGKGYYIHEKGSKPKPDLSVQSIIEESRRLTNAMPGGK-----
Leryth_020735-t1  RNGKSNGKGYYIHEKGSKPKPDLSVQSIIEESRRLTNAMPGGK-----

                       630       640       650       660       670
                  :----|----:----|----:----|----:----|----:----|--
Consensus         ---------------------------PIXVSDQEIVEMILFPVVNEA
Leryth_018504-t1  PHMNSLCYTSWYKFHETNNTFTFQWLQPIVISDQEVVEMILFPVVNEA
PtCHD1            ---------------------------PINITDKEIVEMVLFPVVNEA
PtCHD2            ---------------------------LAKLPEKDIVEMIFFPVVNEA
PtCHD3            ---------------------------LAKLPEKDIVEMIFFPVVNEA
PhCHD             ---------------------------PISVTDQEIVEMILFPVVNEA
Leryth_020735-t2  ---------------------------PIAVSDQEVVDMILFPVVNEA
Leryth_020735-t1  ---------------------------PIAVSDQEVVDMILFPVVNEA

                         680       690       700       710       720
                  --:----|----:----|----:----|----:----|----:----|
Consensus         CRVMDEGIVVRASDLDVASVLGMGFPSYRGGIVFWADLVGAXHIYXSL
Leryth_018504-t1  CRVMDEGIVIRASDLDVASVLGMGFPSYR------------------L
PtCHD1            CRVLDEGVVVRASDLDTASVLGMSFPSYRGGIVFWADLVGPKHVYDSL
PtCHD2            CRVFAEGIAVKAADLDISSLMGMGFPPYRGGIMFWADSLGSKYIYSRL
PtCHD3            CRVFAEGIAVKAADLDIASLMGMGFPPYRGGIMFWADSFGSKYIYSRL
PhCHD             CRVLDEGIVVRASDLDVASVLGMSFPSYRGGIVFWADTVGAGHIYKSL
Leryth_020735-t2  CRVMDEGIVIRASDLDVASVLGMGFPSYRGGIVFWADLVGAGHIYESL
Leryth_020735-t1  CRVMDEGIVIRASDLDVASVLGMGFPSYRGGIVFWADLVGAGHIYESL

                           730       740       750       760
                  ----:----|----:----|----:----|----:----|--
Consensus         KKWSEVYGDFYKPSRFLEERATXGIPLSAPASXSSXSR-SRL
Leryth_018504-t1  KKWSEVYGDFYKPSRFLEQRAAAGIPLSAPASSSSSSRSSRL
PtCHD1            KKWSQRFGDFYKPSKFLEERATGGIPLSAPASSSSGSR-SRM
PtCHD2            EEWSKTYGEFFKPCAFLAERAAKGAPLSSPVDQAK----SRL
PtCHD3            EEWSKTYGEFFEPCAFLAERGAKGAPLSSPVEQAK----SRL
PhCHD             TKWSELYGNFFKPSRFLEERATKGIALSAPATASSASR-SRM
Leryth_020735-t2  KKWSEVYGDFYKPSRFLEQRATAGIPLSAPTSTSSSSR-SRL
```

```
Leryth_020735-t1 KKWSEVYGDFYKPSRFLEQRATAGIPLLDSKIFS--------
```

Supplemental Figure S4. Amino acid sequence comparison of putative *L. officinale* cinnamoyl-CoA hydratase (CHD; Leryth_018504, and Leryth_020735) gene recovered in our coexpression network with gene annotated as CHD in the genome and the characterized CHD genes from *Petunia hybrida* (PhCHD) and *Poplar trichocarpa* (PtCHD1, PtCHD2 and PtCHD3). The amino acid alignments were computed using the MUSCLE algorithm implemented in Geneious. The pink highlighted sequence is the peroxisomal targeting sequence I (PTS I), predicted by PSORT (https://www.psort.org/). The predictive hydratase and dehydrogenase domains are highlighted by yellow and green colours, respectively. These domains were predicted by ProSite (https://prosite.expasy.org/scanprosite/).

```
                              10        20        30        40
                   ----:----|----:----|----:----|----:----|----:---
Consensus          MYMLMEKA-------------------------IXRQRVLLZHLRPSX
Leryth_003297-t2   ----MEKA-------------------------INRQRVLLGHLQPSS
Leryth_016775-t1   MYMLHKKCYGLSPIIGHNYNLDVIGPVIDVGILVKRVKAMVEFAKDPS
Leryth_002701-t1   ----MEKA-------------------------IQRQRVLLQHLRPS-
Leryth_002701-t3   ----MEKA-------------------------IQRQRVLLQHLRPS-
Leryth_002701-t2   ----MEKA-------------------------IQRQRVLLQHLRPS-
Leryth_016775-t2   ----MEKA-------------------------IERQRVLLHHLKPCH
Leryth_016775-t3   ----MEKA-------------------------IERQRVLLHHLKPCH
PtKAT2             ----MEKA-------------------------INRQRVLLEHLRPSS
PtKAT3             ----MEKA-------------------------SNRQRVLLGHLRSSS
PtKAT1             ----MEKA-------------------------FNRQKVLLAHLQPT-
PhKAT1             ----MEKA-------------------------IQRQRVLLEHLQPIR

                    50        60        70        80        90
                   -|----:----|----:----|----:----|----:----|----:-
Consensus          ----LNNM---DSNLSASVCAAGDSASYQRTSVYGDDVVIVAAYRSPL
Leryth_003297-t2   SDD-HQNY---ISNVSPSICLAEGG---KLKAPSGDDVVIVAAYRTPL
Leryth_016775-t1   STIHEKELLLGKENQFASVCAAGDSASYQRTSVYGDDVVIVAAYRSPL
Leryth_002701-t1   ----LNNM---DSNLSASVCAAGDSASYQRTSVYGDDVVIVAAYRSPL
Leryth_002701-t3   ----LNNM---DSNLSASVCAAGDSASYQRTSVYGDDVVIVAAYRSPL
Leryth_002701-t2   ----LNNM---DSNLSASVCAAGDSASYQRTSVYGDDVVIVAAYRSPL
Leryth_016775-t2   TSSPLENI---DSSLSASVCAAGDSASYQRTSVYGDDVVIVAAYRSPL
Leryth_016775-t3   TSSPLENI---DSSLSASVCAAGDSASYQRTSVYGDDVVIVAAYRSPL
PtKAT2             ----SHNF---ESTLSASACLAGDSAAYQRTSAYGDDVVIVAAYRTPQ
PtKAT3             S---YNNH---ESSLSASACLAGGS------AACGDDVVVVAAYRTPL
PtKAT1             ------SP---SPLLSASICAAGDSAAYHRTAAFGDDIVIVAAYRTAI
PhKAT1             HH--THDH---SSSLTTSICAAGDSAAYQRTAAFGDDVVIVAAYRTAI

                      100       110       120       130       140
                   ---|----:----|----:----|----:----|----:----|----
Consensus          CKAKRGGLKDTYXDDLLAPVLRALIEKTNLDPSEVGDIVVGSVLAPGS
Leryth_003297-t2   CKAKRGGFKDTHPDDLLAAVLKALIDKTHVKPTEVGDVIVGTVLAPGS
Leryth_016775-t1   CKAKRGGLKDTYPDDLLAPVLRALIEKTNVDPSEIGDIVVGSVLAAGS
Leryth_002701-t1   CKAKRGGLKDTYADDLLAPVLRALIEKTNLDPSEVGDIIVGSVLAPGS
Leryth_002701-t3   CKAKRGGLKDTYADDLLAPVLRALIEKTNLDPSEVGDIIVGSVLAPGS
Leryth_002701-t2   CKAKRGGLKDTYADDLLAPVLRALIEKTNLDPSEVGDIIVGSVLAPGS
Leryth_016775-t2   CKAKRGGLKDTYPDDLLAPVLRALIEKTNVDPSEIGDIVVGSVLAAGS
Leryth_016775-t3   CKAKRGGLKDTYPDDLLAPVLRALIEKTNVDPSEIGDIVVGSVLAAGS
PtKAT2             CKSKRGGFKDTHADDLLAPVLKALIEKTNLDPREVGDIVVGSVLAPGS
PtKAT3             CKSKRGGFKDTHADDLLAPVLKALIEKTNLDAREVGDIVVGSSLAPGS
PtKAT1             CKARRGGFKDTFPDDLLGFVLKAVVEKTNVNPSEVGDIVVGTVLAPGS
PhKAT1             CKSKRGGFKDTLSDDLLAPVLKAVIEKTNLDPKEVGDIVVGTVLAPGS

                        150       160       170       180       190
                   :----|----:----|----:----|----:----|----:----|--
Consensus          QRASECRMAAFYAGFPETVPVRTVNRQCSSGLQAVADVAASIKAGFYD
Leryth_003297-t2   LRAIEFRMGQFYAGFPATVPIRTVNRQCSSGLQAVADVAAYIKAGYYD
Leryth_016775-t1   QRASECRMAAFYAGFPDTVPVRTVNRQCSSGLQAVADVAASIKAGFYD
Leryth_002701-t1   QRASECRMAAFYAGFPETVPVRTVNRQCSSGLQAVADVAAAIRAGFYD
Leryth_002701-t3   QRASECRMAAFYAGFPETVPVRTVNRQCSSGLQAVADVAAAIRAGFYD
Leryth_002701-t2   QRASECRMAAFYAGFPETVPVRTVNRQCSSGLQAVADVAAAIRAGFYD
Leryth_016775-t2   QRASECRMAAFYAGFPDTVPVRTVNRQCSSGLQAVADVAASIKAGFYD
Leryth_016775-t3   QRASECRMAAFYAGFPDTVPVRTVNRQCSSGLQAVADVAASIKAGFYD
PtKAT2             QRASECRMAAFYAGFPETVPIRTVNRQCSSGLQAVADVAASIKAGFYE
PtKAT3             QRASECRMAALYAGFPETVPIRTVNRKCSSGLQAVADVAASIKAGFYE
PtKAT1             ERAIECRMAAFYAGFPDTVPLRTVNRQCSSGLQAVADVAASIKAGYYD
```

```
PhKAT1             IRAMECRMAAFYAGFPETVPIRTVNRQCSSGLQAVADVAASIKAGFYD

                          200       210       220       230       240
                   --:----|----:----|----:----|----:----|----:----|
Consensus          IGIGAGLESMTXNPMAWEGSVNPRVKSMAQAQDCLLPMGITSENVAHR
Leryth_003297-t2   IGIGAGVECMSIDTISNISNPNPKAQSFPQAQDCLLPMGVTSENVAQR
Leryth_016775-t1   IGIGAGLESMTTNPMAWEGSVNPRVKSMAQAQDCLLPMGITSENVAHR
Leryth_002701-t1   IGIGAGLESMTANPMAWEGSVNPRVKSMAQAQDCLLPMGITSENVAHR
Leryth_002701-t3   IGIGAGLESMTANPMAWEGSVNPRVKSMAQAQDCLLPMGITSENVAHR
Leryth_002701-t2   IGIGAGLESMTANPMAWEGSVNPRVKSMAQAQDCLLPMGITSENVAHR
Leryth_016775-t2   IGIGAGLESMTTNPMAWEGSVNPRVKSMAQAQDCLLPMGITSENVAHR
Leryth_016775-t3   IGIGAGLESMTTNPMAWEGSVNPRVKSMAQAQDCLLPMGITSENVAHR
PtKAT2             IGIGAGLESMTINQMSWDGDVNPKLKNFQKAQDCLLPMGITSENVAHR
PtKAT3             IGIGAGLESMTVNSRAWVGDVNPKVKRFQEAQDCLLPMGVTSENVAHR
PtKAT1             IGIAAGLECMTVNGIRSVPQVNPRVEAFQQARDCLLPMGLTSENVAQR
PhKAT1             IGIGAGLELMTVDNIGRVQQRNTKVDTFAQARDCLLPMGITSENVAQR

                            250       260       270       280
                   ----:----|----:----|----:----|----:----|----:---
Consensus          FGVSRQEQDQAAVNSHRKAAAATASGKFKDEIIPVNTKIVDPKTGDEK
Leryth_003297-t2   YGVTREEQDQAAVISHKRAAAASASGKFIDEIIPVSTKIVDPKTGEIK
Leryth_016775-t1   FGVSRQEQDQAAVNSHRKAAAATASGKFKDEIIPVNTKIVDPKTGDEK
Leryth_002701-t1   FGVSRQEQDQAAVNSHRKAAAATASGKFKDEIIPVNTKIVDPKTGDEK
Leryth_002701-t3   FGVSRQEQDQAAVNSHRKAAAATASGKFKDEIIPVNTKIVDPKTGDEK
Leryth_002701-t2   FGVSRQEQDQAAVC-----------------------------------
Leryth_016775-t2   FGVSRQEQDQAAVNSHRKAAAATASGKFKDEIIPVNTKIVDPKTGDEK
Leryth_016775-t3   FGVSRQEQDQAAVNSHRKAAAATASGKFKDEIIPVNTKIVDPKTGDEK
PtKAT2             FGVTRQEQDQAAVDSHRKAAAATASGRFKDEIIPVATKIVDPKTGDEK
PtKAT3             FSVTRQEQDQAAVDSHRKAAAATASGRFKDEIIPVATKIADPKTGDEK
PtKAT1             YGVTRQDQDQAAVDSHRKAAAARASGKFKDEIIPVTTKIVDPETGIQK
PhKAT1             FGVTRLEQDQAAVNSHQRAAAATASGKFKDEIIPVLTKIVDPQTGKEK

                    290       300       310       320       330
                   -|----:----|----:----|----:----|----:----|----:-
Consensus          PVTISVDDGIRPNASVADLAKLKPVFKKDGTTTAGNSSQVSDGAGAVL
Leryth_003297-t2   PVIIAADDGIRPTTNLADLAKLKPAFSKNGTTTAGNASQVTDGAGGVL
Leryth_016775-t1   PVTISIDDGIRPNATVSDLAKLKPVFKKDGTTTAGNSSQVSDGAGAVL
Leryth_002701-t1   PVTISVDDGIRPNASVADLAKLKPVFKKDGTTTAGNSSQVSDGAGAVL
Leryth_002701-t3   PVTISVDDGIRPNASVADLAKLKPVFKKDGTTTAGNSSQVSDGAGAVL
Leryth_002701-t2   ------------------------------------------------
Leryth_016775-t2   PVTISIDDGIRPNATVSDLAKLKPVFKKDGTTTAGNSSQVSDGAGAVL
Leryth_016775-t3   PVTISIDDGIRPNATVSDLAKLKPVFKKDGTTTAGNSSQVSDGAGAVL
PtKAT2             PIVVSVDDGIRPETSVAGLGKLKTVFKKDGTTTAGNSSQISDGAGAVL
PtKAT3             PIIVSVDDGIRPNTSLADLGKLKAVFRKDGTTTAGNSSQISDGAAAVL
PtKAT1             VITISEDDGIRPNSNLAELSRLKPAFKKDGSTTAGNASQVSDGAGAVL
PhKAT1             PVVISVDDGIRPNTNLTSLGKLKPAFKNDGTTTAGNASQVSDGAAAVL

                      340       350       360       370       380
                   ---|----:----|----:----|----:----|----:----|----
Consensus          LMKRSVAMKKGLPXLGVFRTFAAVGVDPAIMGVGPAVAIPAAVKSAGL
Leryth_003297-t2   LMKRSVAIKKGLPILGVWRSFEAVGVEPGVMGIGPAVAIPAAVKSAGL
Leryth_016775-t1   LMKRSVAMKKGLPVLGVFRTFAAVGVDPAIMGVGPAVAIPAAVKSAGL
Leryth_002701-t1   LMKRSVAMQKGLPVLGVFR-----------------------------
Leryth_002701-t3   LMKRSVAMQKGLPVLGVFR-----------------------------
Leryth_002701-t2   ------------------------------------------------
Leryth_016775-t2   LMKRSVAMKKGLPVLGVFRTFAAVGVDPAIMGVGPAVAIPAAVKSAGL
Leryth_016775-t3   LMKRSVAMKKGLPVLGVFRTFAAVGVDPAIMGVGPAVAIPAAVKSAGL
PtKAT2             LMKRSVAVQKGLPILGVFRTFAAVGVDPAIMGVGPAVAIPAAVKAAGL
```

```
PtKAT3              LMKRSVAMRKGLPILGVFRTFVVVGVDPAIMGVGPAVAIPAAVKAAGL
PtKAT1              LMKRSLAAQKGLPILGVFRSFAAVGVDPAVMGVGPAAAIPAAVQAAGL
PhKAT1              LMKRSVAMKKGLPILGVFRSFAAVGVDPAVMGIGPAVAIPPAVKSAGL

                         390       400       410       420       430
                    :----|----:----|----:----|----:----|----:----|--
Consensus           ELEDIDLFEINEAFASQFVYCQKKLELDPEKINVNGGAMAIGHPLGAT
Leryth_003297-t2    ELDDISLFEINEAFASQYVYCCKKLNLDPERVNVNGGAMALGHPLGAT
Leryth_016775-t1    ELEDIDLFEINEAFASQFVYCQKKLELDPEKINVNGGAMAIGHPLGAT
Leryth_002701-t1    ------------------------------------------------
Leryth_002701-t3    ------------------------------------------------
Leryth_002701-t2    ------------------------------------------------
Leryth_016775-t2    ELEDIDLFEINEAFASQFVYCQKKLELDPEKINVNGGAMAIGHPLGAT
Leryth_016775-t3    ELEDIDLFEINEVI----------------------------------
PtKAT2              ELEDIDLFEINEAFASQFVYCRKKLELDPQKINVNGGAMAIGHPLGAT
PtKAT3              ELEDIDLFEINEAFASQFVYCCKKLELDLQKINVNGGAIAIGHPLGTT
PtKAT1              ELNDIDLFEINEAFASQYVYCIKKLELDPEKVNVNGGAIALGHPLGAT
PhKAT1              DLDDIDLYEINEAFASQFVYCQKKLNLDPEKVNVNGGAMALGHPLGAT

                           440       450       460       470       480
                    --:----|----:----|----:----|----:----|----:----|
Consensus           GARCVATLLHEMKRRGXDCRFGVXSMCIGXGMGAAAVFERGDGCDELC
Leryth_003297-t2    GARCVATLLHEMKRRGKDCRFGVISMCIGSGMGAAAVFERGDF-----
Leryth_016775-t1    GARCVATLLHEMKRRGRDCRFGVVSMCIGTGMGAAAVFERGDSCDELR
Leryth_002701-t1    ------------------------------------------------
Leryth_002701-t3    ------------------------------------------------
Leryth_002701-t2    ------------------------------------------------
Leryth_016775-t2    GN----------------------------------------------
Leryth_016775-t3    ------------------------------------------------
PtKAT2              GARCVATLLHEMKRRGRDCRFGVVSMCIGTGMGAAAVFERGDGCDELC
PtKAT3              GARCVATILHEMKRRGRDCRFGVVSMCIGTGMGAAAVFERGDGCDELC
PtKAT1              GARCVGTLLNEMKRRGKDSRFGVISMCIGSGMGAAAVFERGDCVDALC
PhKAT1              GARCVATLLHEMKRRGKDCRFGVISMCIGSGMGAAAVFERGDAVDDLC

                             490
                    ----:----|----:--
Consensus           N-ARKVESNNLLSKDAR
Leryth_003297-t2    -----------------
Leryth_016775-t1    N-ARKIDVNNQLSKDAR
Leryth_002701-t1    -----------------
Leryth_002701-t3    -----------------
Leryth_002701-t2    -----------------
Leryth_016775-t2    -----------------
Leryth_016775-t3    -----------------
PtKAT2              NGGRKVESNNLLSRDSR
PtKAT3              N-ARKVESNNLLSKDSR
PtKAT1              N-VRKVGSNDLLSKDAR
PhKAT1              N-ARVSNNNSFLSKDAK
```

Supplemental Figure S5. Amino acid sequence comparison of putative *L. officinale* 3-ketoacyl-CoA thiolase (KAT: Leryth_003297) gene recovered in our coexpression network, with genes annotated as KAT in the genome and the characterized KAT genes from *Petunia hybrida* (PhKAT1) and *Poplar trichocarpa* (PtKAT1, PtKAT2 and PtKAT3). The amino acid alignments were computed using the MUSCLE algorithm implemented in Geneious. The pink highlighted sequence is the peroxisomal

targeting sequence I (PTS I), predicted by PSORT (https://www.psort.org/). The putative regions involved in thiolase activity are highlighted by yellow colours. These domains were predicted by ProSite (https://prosite.expasy.org/scanprosite/).

```
                             10        20        30        40
                     ----:----|----:----|----:----|----:----|----:---
Consensus            MDX--------XXX-ASSKTXIIDXPLHXLGFEFDELSPTXXXGRLXX
PhTE1                MKP-----PPLSSSAAGIKIREIDIPLHEIGFEYVEITPHKISGRLHL
AtDNAT2              MDP-------------KSPEFIIDQPLKILGFVFDELSATRVSGHLTL
Leryth_026039-t1     MDSTRKPEAQSSSSSSSNKTQILDSPLHTLGFEIEELSSNKVTGKLIV
AtDNAT1              MDS------------ASSNTKAIDPPLHMLGFEFDELSPTRITGRLPV

                       50        60        70        80        90
                     -|----:----|----:----|----:----|----:----|----:-
Consensus            TEKCCQPFKVLHGGVSALIAESLASMGAHIASGFKRVAGIHLSIXHLK
PhTE1                TERCCQPFKVLHGGISALIAESLASMGAHIASGFTRVAGVHLSIHHLK
AtDNAT2              TEKCCQPFKVLHGGVSALIAEALASLGAGIASGFKRVAGIHLSIHHLR
Leryth_026039-t1     NEKCLQPFRVLHGGVSAVIAEGLASMGAHIASGWRRVAGIHLSINHIK
AtDNAT1              SPVCCQPFKVLHGGVSALIAESLASMGAHMASGFKRVAGIQLSINHLK

                        100       110       120       130       140
                     ---|----:----|----:----|----:----|----:----|----
Consensus            SAXLGDLVFAEATPVSVGKTIQVWEVRLWKXTKXEEX-NKIXISSSRV
PhTE1                SAQLGDLVIAEAAPVNVGQSIQVWEVCLWKIYESNEEEKKTLIASSRV
AtDNAT2              PAALGEIVFAESFPVSVGKNIQVWEVRLWKAKKTETPDNKIMVSTSRV
Leryth_026039-t1     SATLGDLVVAEATPVSVGKTIQIWEVRLWKVTKEEE---KSMISSSRV
AtDNAT1              SADLGDLVFAEATPVSTGKTIQVWEVKLWKTTQKDKA-NKILISSSRV

                         150       160       170
                     :----|----:----|----:----|
Consensus            TLXCNLPXPDNAKDAAXXLKKX-AKL
PhTE1                TLKTNMFVPENAKDAAVVLKKY-AKL
AtDNAT2              TLFCGLPIPDHVKDAPDELKKVISKL
Leryth_026039-t1     TLLCNLPVPQDS-DAAPNLRKY-AKL
AtDNAT1              TLICNLPIPDNAKDAANMLKMV-AKL
```

Supplemental Figure S6. Amino acid sequence comparison of putative *L. officinale* thioesterase (TE: Leryth_026039) gene recovered in our coexpression network with the characterised TE genes from *Petunia hybrida* (PhTE1) and *Arabidopsis thaliana* (AtDNAT1 and AtDNAT2). The amino acid alignment was computed using the MUSCLE algorithm implemented in Geneious. The pink highlighted sequence is the peroxisomal targeting sequence I (PTS I), needed por peroxisomal localization.

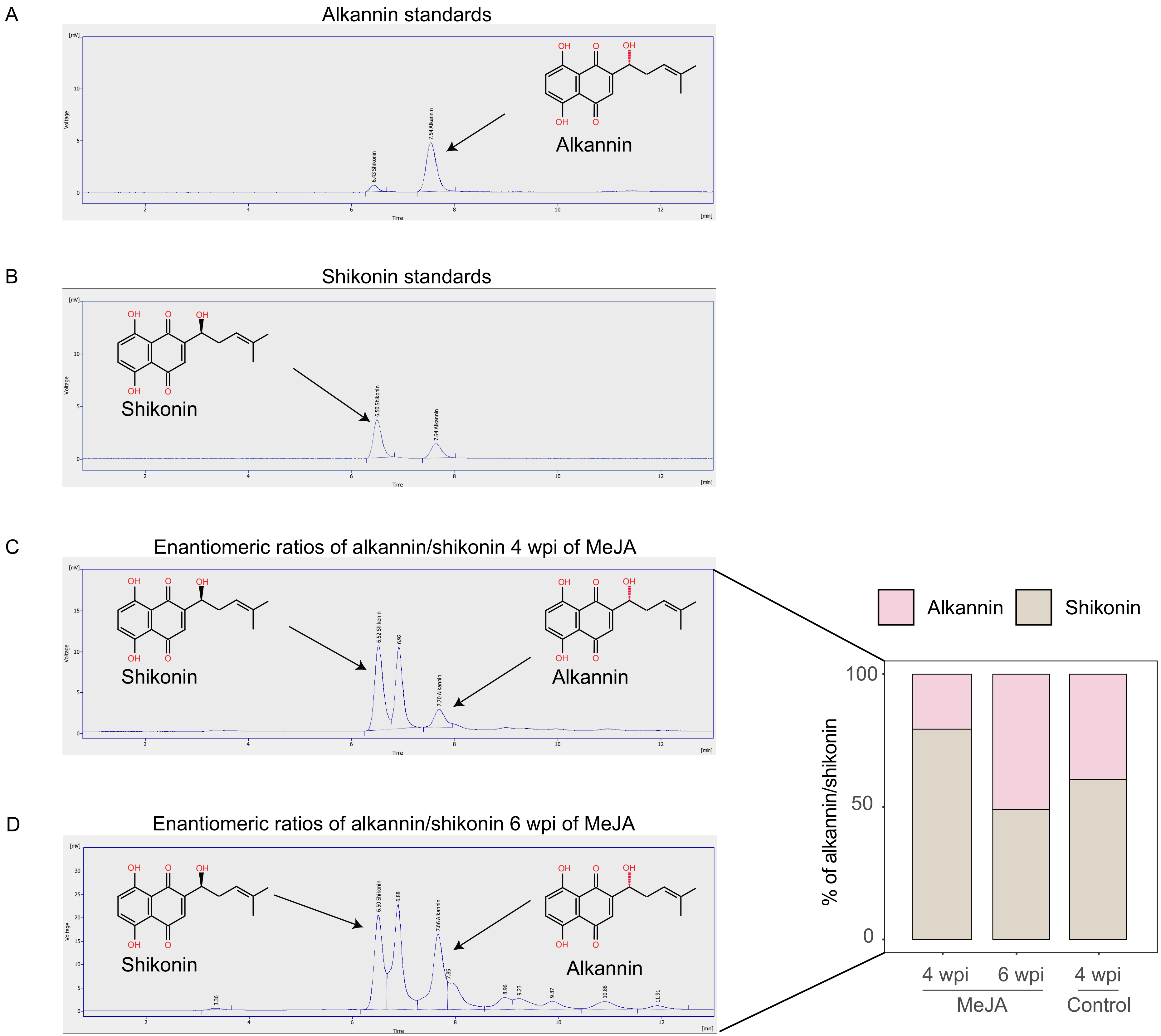

Supplemental Figure S7. Enantiomeric ratios of alkannin/shikonin (A/S) in MeJA treated plants of *L. officinale*. HPLC chromatogram of **A)** alkannin standards **B)** shikonin standards C) enantiomeric ratios of alkannin/shikonin at 4 wpi and D) 6 wpi. The inset stacked bar graphs represent percentage composition of alkannin:shikonin in plants of MeJA treatment.

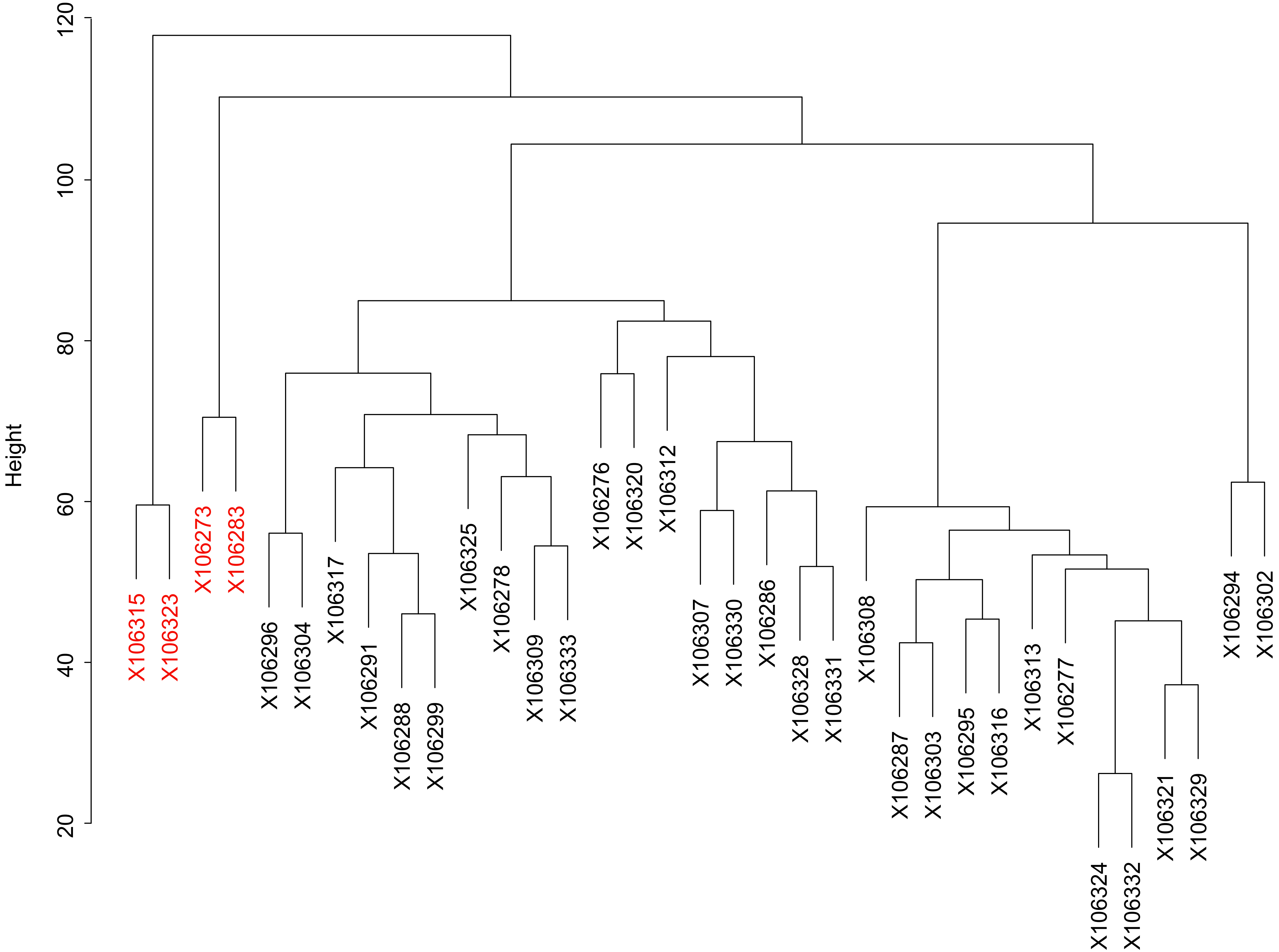

Supplemental Figure S8. Clustering of samples to detect outliers. Samples highlighted in red were removed before coexpression analysis.
