# Supplementary material for: Dynamics of alkannin/shikonin biosynthesis in response to jasmonate and salicylic acid in Lithospermum officinale
Source: Sci Rep. 2022 Oct 12;12:17093. doi: 10.1038/s41598-022-21322-0 (PMC9554848; doi:10.1038/s41598-022-21322-0)
Supplement: Supplementary file 2 — Supplementary Information. [file 41598_2022_21322_MOESM2_ESM.docx]

**Sample preparation and chiral-HPLC analysis**

For the determination of the enantiomeric ratio of A/S derivatives in the samples, root extracts were hydrolyzed according to the procedure described by Tappeiner et. al, 2014 (Tappeiner et al., 2014). Briefly, 1 mL of methanolic roots extract hydrolyzed with 60 mL of NaOH (1N) for 3h under stirring at room temperature. After acidification with HCl 37%, extraction of the hydrolyzed Alkannin and shikonin was performed with chloroform. Finally, chloroform was evaporated and the solid residue was dissolved in hexane: 2-propanol 9:1 v/v before analysis.

The chromatographic analysis took place at a Chiralcel ODH0CE-LJ110 column (5 μM; 250 × 4.6mm), according to Tappeiner et. al, 2014 analysis method slightly modified. Measurements were performed at a liquid chromatograph system, equipped with a UV–Vis detector (Fasma 500) set at 520 nm and a Marathon III HPLC pump (all from Rigas Labs, Thessaloniki, Greece). The isocratic elution program consisted of n-hexane and 2-propanol at a 65:35 ratio with a flow rate of 0.8 mL/min, while the elution time was 15 min. The retention time of each enantiomer was determined by A/S standards (commercial and purified ones by column chromatography and recrystallization) that were evaluated for their enantiomeric ratio estimation by polarimetry studies, chiral HPLC-analyses and circular dichroism spectra by A. Assimopoulou.

Tappeiner, J., Vasiliou, A., Ganzera, M., Fessas, D., Stuppner, H., Papageorgiou, V.P., Assimopoulou, A.N., 2014. Quantitative determination of alkannins and shikonins in endemic Mediterranean *Alkanna* species: Quantitative determination of alkannins and shikonins. Biomed. Chromatogr. 28, 923–933. https://doi.org/10.1002/bmc.3096
